# Transcriptomic responses of lung mesenchymal cells during pneumonia

**DOI:** 10.1172/jci.insight.177084

**Published:** 2025-02-25

**Authors:** Alicia M. Soucy, Jourdan E. Brune, Archana Jayaraman, Anukul T. Shenoy, Filiz T. Korkmaz, Neelou S. Etesami, Bradley E. Hiller, Ian M.C. Martin, Wesley N. Goltry, Catherine T. Ha, Nicholas A. Crossland, Joshua D. Campbell, Thomas G. Beach, Katrina E. Traber, Matthew R. Jones, Lee J. Quinton, Markus Bosmann, Charles W. Frevert, Joseph P. Mizgerd

**Affiliations:** 1 Pulmonary Center, Boston University Chobanian & Avedisian School of Medicine, Boston, Massachusetts, USA.; 2 Department of Comparative Medicine, University of Washington School of Medicine, Seattle, Washington, USA.; 3 Center for Lung Biology, University of Washington, Seattle, Washington, USA.; 4 Department of Microbiology and Immunology, University of Michigan Medical School, Ann Arbor, Michigan, USA.; 5 University of Massachusetts Chan Medical School, Worcester, Massachusetts, USA.; 6 National Emerging Infectious Diseases Laboratory, Boston University, Boston, Massachusetts, USA.; 7 Department of Pathology and Laboratory Medicine;; 8 Department of Virology, Immunology, & Microbiology; and; 9 Department of Medicine, Boston University Chobanian & Avedisian School of Medicine, Boston, Massachusetts, USA.; 10 Banner Sun Health Research Institute Brain and Body Donation Program, Sun City, Arizona, USA.; 11 Center for Thrombosis and Hemostasis, University Medical Center of the Johannes Gutenberg-University, Mainz, Germany.; 12 Department of Medicine, Division of Pulmonary, Critical Care and Sleep Medicine, University of Washington, Seattle, Washington, USA.; 13 Department of Biochemistry and Cell Biology, Boston University Chobanian & Avedisian School of Medicine, Boston, Massachusetts, USA.

**Keywords:** Immunology, Pulmonology, Bacterial infections, Cellular immune response, Innate immunity

## Abstract

The role of mesenchymal cells during respiratory infection is not well defined, including whether, which, and how the different types of mesenchymal cells respond. We collected all mesenchymal cells from lung single-cell suspensions of mice that were naive (after receiving only saline vehicle), pneumonic (after intratracheal instillation of pneumococcus 24 hours previously), or resolved from infection (after nonlethal pneumococcal infections 6 weeks previously) and performed single-cell RNA sequencing. Cells clustered into 5 well-separated groups based on their transcriptomes: matrix fibroblasts, myofibroblasts, pericytes, smooth muscle cells, and mesothelial cells. Fibroblasts were the most abundant and could be further segregated into *Pdgfra^+^Npnt^+^Ces1d^+^Col13a1^+^* alveolar fibroblasts and *Cd9^+^Pi16^+^Sca1^+^Col14a1^+^* adventitial fibroblasts. The cells from naive and resolved groups overlapped in dimension reduction plots, suggesting the mesenchymal cells returned to baseline transcriptomes after resolution. During pneumonia, all mesenchymal cells responded with altered transcriptomes, revealing a core response that had been conserved across cell types as well as distinct mesenchymal cell type–specific responses. The different subsets of fibroblasts induced similar gene sets, but the alveolar fibroblasts responded more strongly than the adventitial fibroblasts. These data demonstrated diverse and specialized immune activities of lung mesenchymal cells during pneumonia.

## Abstract 2

Pneumonia induces transcriptomic changes throughout lung mesenchymal cells, including shared core and cell-type-specific responses, after which cells return in recovered lungs to their initial state.

## Introduction

Pneumonia remains a serious public health burden globally. Even before the COVID-19 pandemic, pneumonia in the United States was the most common reason for hospitalization of children under the age of 9 and accounted for nearly half of the infectious disease–related hospitalizations and deaths among adults over the age of 65 (1–4). Identifying host mechanisms protecting healthy young adults, who have lower incidence and severity of respiratory infection, is a priority in pneumonia research (2, 5). Most lung infections require recruited leukocytes for successful defense, as both microbe killers and important modifiers of the tissue response (6–9). After recovery from prior infections, the lungs have greatly improved defense abilities, due in part to resident memory lymphocytes that populate experienced but not naive lungs (10–14). Additionally, cells such as alveolar macrophages, which are present in both naive and experienced lungs, display altered transcriptomes, surface markers, and metabolomes after recovery from prior respiratory infections (15–19). Better understanding of lung immune responses to infection will guide development of means for assessing and bolstering host defense against pneumonia (2, 5).

The lungs contain many diverse mesenchymal cells, but their possible immune role during infection is only beginning to be explored. In response to severe influenza infection, fibroblasts produce extracellular matrix-remodeling (ECM-remodeling) enzymes and inflammatory cytokines to modify the lung microenvironment and promote immune cell infiltration (20). After experimental vaccinations, IL-33 from fibroblasts is necessary for CD8^+^ T cell recruitment and memory cell formation in the lungs (21), and lung defense against *Klebsiella pneumoniae* pneumonia requires IL-17R signaling in fibroblasts (22). In response to inflammatory mediators such as LPS and TNF, pericytes can instruct neutrophils where to go using pattern recognition receptors, adhesion molecules, cytokines, and motility programs (23, 24). Roles of myofibroblasts, smooth muscle cells, and mesothelial cells during infection are the least understood, but they express pattern recognition receptors and release pro-inflammatory mediators including IL-1, IL-6, and TNF (25–28). Thus, mesenchymal cells have the means to contribute to integrated pulmonary immune responses during pneumonia.

Mesenchymal cells are well positioned to guide recruited leukocytes in the lung. Reconstructed serial transmission electron microscopy sections of alveolar septae show interstitial cells that morphologically resemble fibroblasts making direct contact with endothelial and epithelial cells at preexisting holes in the basal lamina in the absence of inflammation and making contact with migrating neutrophils during pneumococcal pneumonia (29). The emerging recognition of heterogeneity within fibroblasts and other mesenchymal cells in the lungs demands a better understanding of how immune roles are distributed across cellular subsets (30–33). PDGFRα^+^ fibroblasts in the alveolar septae are in direct contact with type 2 alveolar epithelial cells (34), suggesting they may interact with migrating neutrophils during pneumonia. How and which mesenchymal cells contribute to immunity in the infected lungs are still largely speculative. Whether and how mesenchymal cells change in the lung after recovery from pneumonia have yet to be investigated. In this study, we defined mesenchymal cell responses during acute pneumonia and whether the mesenchymal cells present in the recovered lung were different from those in the naive lung. By using single-cell transcriptomics to delineate the heterogeneity of mesenchymal cells in naive, infected, or recovered lungs, we elucidated shared and distinctive cell responses to respiratory infection.

## Results

### Mesenchymal cell types in the mouse lung.

**Figure 1:**
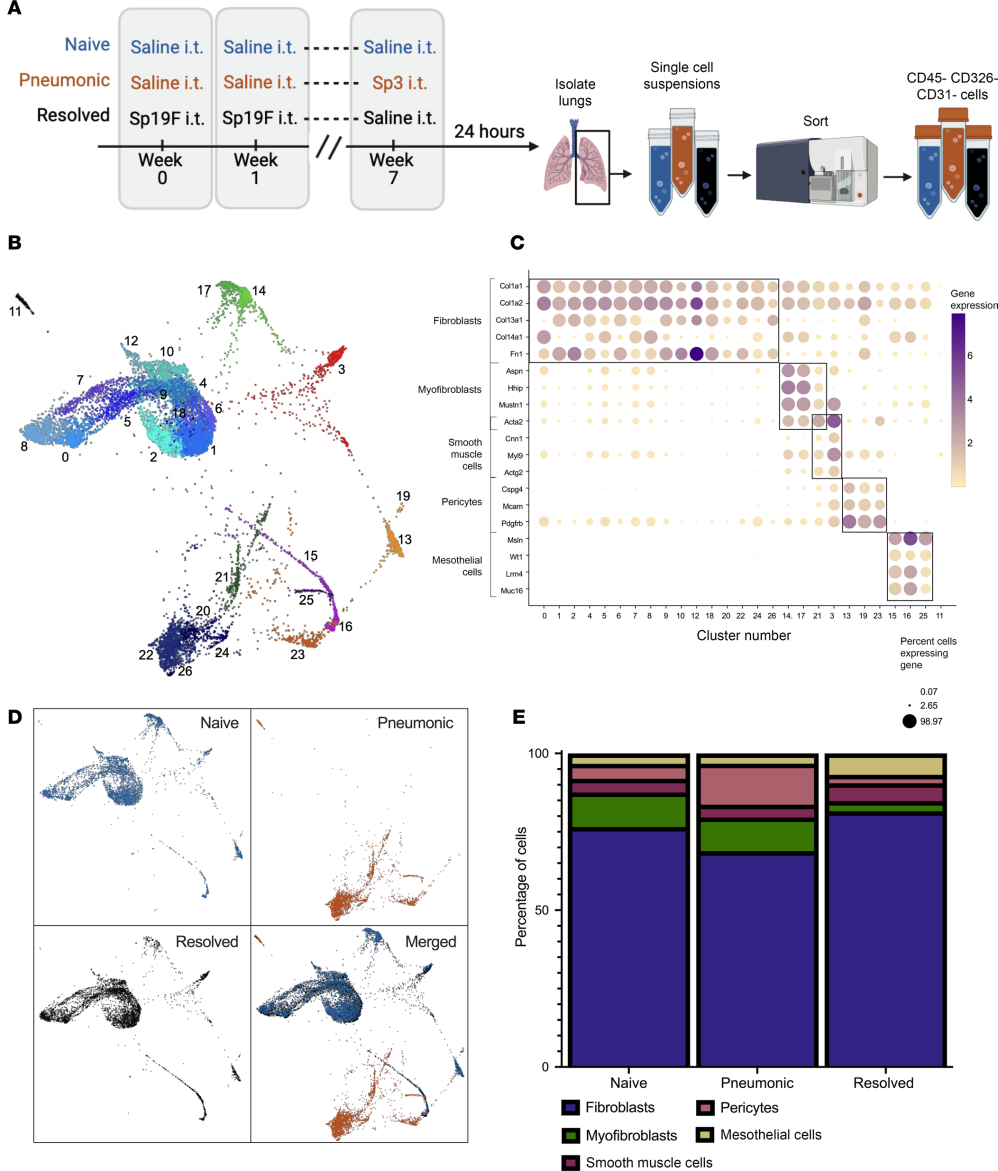
Single-cell RNA-sequencing analysis unveils 5 major mesenchymal cell groups, which all respond to pneumococcal pneumonia. (**A**) A schematic created using BioRender.com shows the timeline of the single-cell RNA-sequencing experiment (3 mice pooled per treatment). (**B**) Using SPRING nearest neighbor plotting, 27 Louvain clusters were discerned among mesenchymal cells (sorted as CD45^–^CD326^–^CD31^–^ cells before single-cell sequencing) after nonmesenchymal cells were removed. (**C**) Each Louvain cluster was assigned to 1 of the 5 mesenchymal cell types based on gene expression specific to each cell type. (**D**) Of the 3 treatment groups, SPRING plots showed that mesenchymal cells from naive (blue) and resolved (black) lungs overlapped, whereas cells from pneumonic (orange) lungs aggregated more closely together in the uniform manifold approximation and projection (UMAP) plots. (**E**) The fractions of lung mesenchymal cells with transcripts defining distinct cell subsets were similar across all 3 treatment groups.

To test whether mesenchymal cells respond to pneumococcal pneumonia, we performed single-cell RNA sequencing (scRNA-Seq) on cells from lung digests using a modified protocol from a previously described method (35). The 3 groups of mice studied differed only regarding whether and when pneumococcus was instilled into their lungs (Figure 1A). The control group, naive, received only the sterile saline vehicle. The active infection group, pneumonic, received pneumococcus 24 hours prior to euthanasia. The recovered group, resolved, had pneumococcus infections a month previously, yielding lungs with no ongoing infection or inflammation but a remodeled resident immune system (2, 12). We used flow cytometry to isolate all cells from the lung that were not leukocytes, epithelial cells, or endothelial cells (i.e., CD45^–^CD326^–^CD31^–^), to compare cell types and transcriptomes between mice that were naive, pneumonic, or resolved (10, 12). scRNA-Seq data were generated with 10x Genomics Chromium and analyzed using SPRING (36).

Analysis of all 14,282 sequenced cells from the 3 groups of mice revealed 31 Louvain clusters (Supplemental Figure 1A; supplemental material available online with this article; https://doi.org/10.1172/jci.insight.177084DS1). A few small clusters expressed genes suggestive of nonmesenchymal populations, such as endothelial cells (*Edn1* and *CD31*, aka *Pecam1*, 416 cells, Supplemental Figure 1, B and C), leukocytes (*CD45*, aka *Ptprc*, 501 cells, Supplemental Figure 1D), and epithelial cells (*Nkx2.1* and *Sftpc*, 406 cells, Supplemental Figure 1, E and F). These cells were removed, and the data were reclustered, resulting in a heterogenous mesenchymal cell population with 27 Louvain clusters across the 3 treatment groups (Figure 1B). Clusters were identified as distinct mesenchymal cell types based on expression of characteristic gene sets, yielding 5 major mesenchymal cell types: fibroblasts (9,872 cells), myofibroblasts (1,008 cells), smooth muscle cells (618 cells), pericytes (744 cells), and mesothelial cells (604 cells) (Figure 1C). Nearly every Louvain cluster was assigned to 1 of those 5 broader cell types. Some studies report small populations of lipofibroblasts in the lung (32, 33, 37). However, none were detected in this study, based on a lack of cells expressing *Adrp*, *Pparg*, *Lpl*, *Fabp1*, *Fabp4*, and *Fabp5* (data not shown). One very small cluster, cluster 11 (113 cells), was not positive for any of the above lineage genes. These cells showed expression of *Myl4*, *Tnni3*, and *Sln*, characteristic of muscle cells (data not shown). These cells were likely the cardiomyocytes that can be found in pulmonary veins of mice (38), and they were not analyzed further.

The largest aggregation of cells in the SPRING plot contained clusters 0, 1, 2, 4, 5, 6, 7, 8, 9, 10, 12, and 18, and the second largest contained clusters 20, 22, 24, and 26 (Figure 1B). We conclude that these are both aggregates of fibroblasts (Figure 1C). Fibroblasts have consistently high expression of ECM proteins, including collagen genes *Col1a1* and *Col1a2* (31), as well as the fibronectin gene, *Fn1* (33). Among lung mesenchymal cells, *Fn1* was expressed by almost all fibroblasts but rarely among other cells (a small subset of pericytes being the exception). Fibroblasts were the most numerous and most heterogeneous mesenchymal cells in the lung, forming 16 Louvain clusters. All fibroblasts had variable expression of *Col13a1* or *Col14a1*, as expected (33). These 16 clusters also expressed at most low levels of the other genes used to define nonfibroblast mesenchymal cell types.

Strong expression of the *Acta2* gene, encoding smooth muscle actin, is a required feature for both myofibroblasts and smooth muscle cells. Myofibroblasts share properties of fibroblasts (ECM synthesis) and smooth muscle cells (contractile function). Clusters 14 and 17 (Figure 1B) aggregated in the plot and were ascribed to be myofibroblasts based on strong expression of *Acta2* plus *Aspn*, *Mustn1*, and *Hhip* (33, 39–42). Cluster 3 was separate (Figure 1B) and inferred to be smooth muscle cells based on *Acta2* plus *Cnn1*, *Myl9*, and *Actg2* (43, 44). Louvain cluster 21 expressed *Acta2* and separated into 2 distinctly appreciable aggregates of cells in the plot (Figure 1B); both myofibroblast and smooth muscle genes were expressed in this Louvain cluster (Figure 1C), so we interpreted this to include both cell types.

Pericytes can be identified by robust expression of *Cspg4* (also known as *Ng2*) plus *Pdgfrb* (33, 45). Additionally, these cells expressed *Mcam* (also known as CD146), which agrees with the precedent for pericytes (33, 46). Pericytes in the lung are heterogeneous based on both Louvain clustering and SPRING plot.

*Msln* encodes the protein mesothelin, which is highly expressed by mesothelial cells, as is *Muc16* (41, 47–49). The *Wt1* gene is important in mesothelial cells’ plasticity (41, 50). All these genes can be expressed by nonmesenchymal cells, but the present data suggest that they are restricted to mesothelial cells within the mesenchymal pool (Figure 1C). *Lrrn4* is reported to be specific to primary mesothelial cells (41, 48), which is also reflected here. This gene signature was thereby used to identify clusters 15, 16, and 25 as mesothelial cells in this dataset.

### Mesenchymal cells in recovered lungs match those of naive mice.

SPRING plots showed that mesenchymal cells from naive and resolved lungs overlapped, suggesting they shared similar cell types, distributions, and transcriptomes (Figure 1, D and E). This contrasts with leukocyte populations in the lungs, which have altered phenotypes, distributions, and transcriptomes in naive versus resolved lungs of mice with pneumococcus histories matched to the current study (10, 12, 15). There were no changes observed in the composition or resting transcriptomes of mesenchymal cells after pneumonia recovery. We postulate that mesenchymal cells return to a preinfection baseline after pneumonia recovery.

### Mesenchymal cell responses during pneumonia.

During pneumonia, the fractions of lung mesenchymal cells with transcripts defining each of the distinct cell subtypes were similar to naive lungs, apart from a possible expansion of pericytes (Figure 1E). However, the transcriptomes of these cells were universally altered, for all mesenchymal cell types. Clusters of mesenchymal cells in the plots from infected lungs aggregated more closely to each other than they did in the plots from naive or resolved lungs (Figure 1D). There was no overlap of mesenchymal cells from pneumonic lungs with those from the naive or resolved groups (Figure 1D). The cells from the infected lungs were still identifiable by their subtype-specific genes (Figure 1C), but their segregation from respective uninfected counterparts, and their congregation (irrespective of identities) in the SPRING plot suggest that infection-induced changes make these cell types more similar to each other at the transcriptome level. These data suggest that all mesenchymal cells responded to acute pneumonia and that a shared response across cell types during pneumonia may render these distinct cell types more transcriptomically similar.

**Figure 2:**
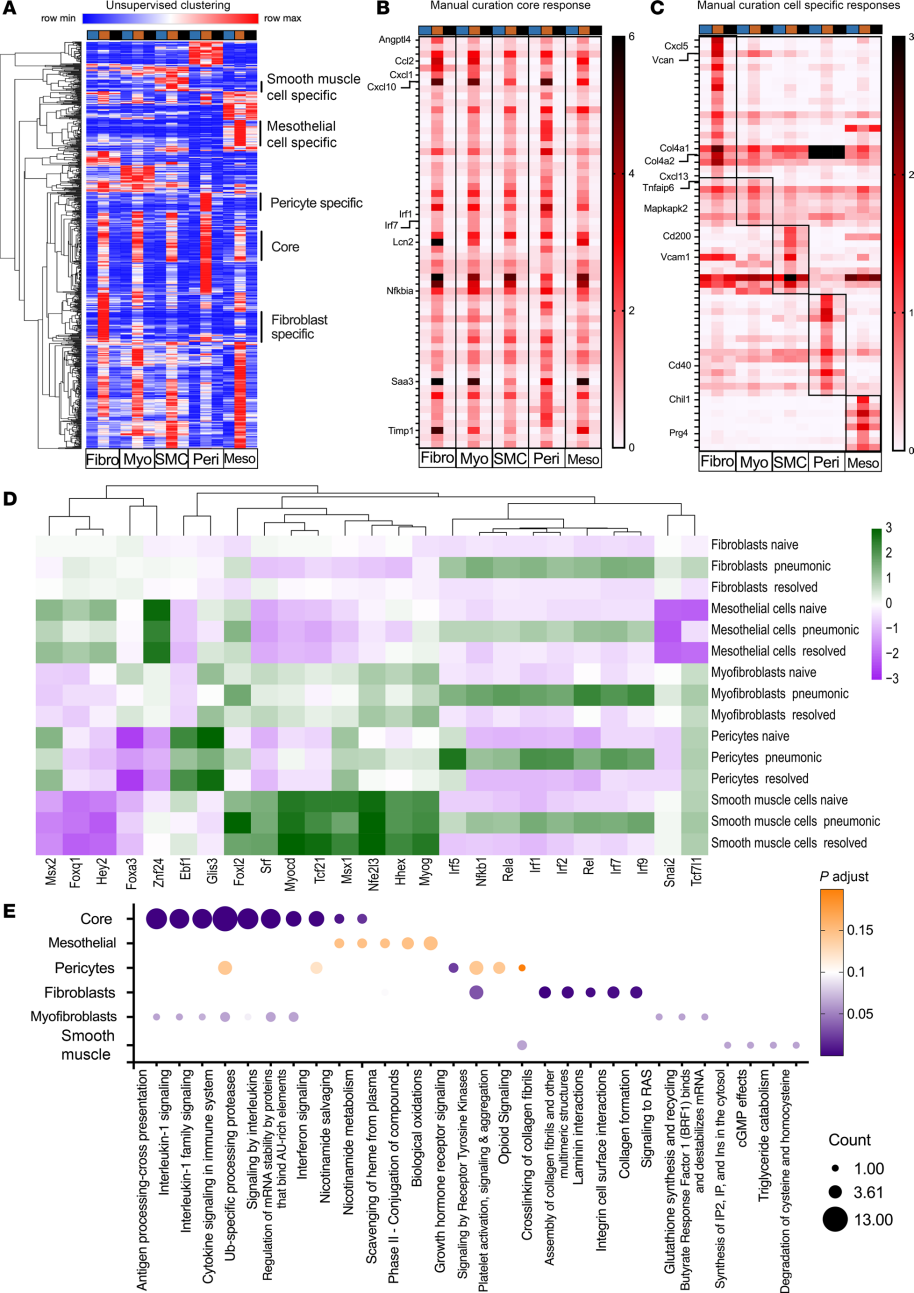
Single-cell RNA-sequencing analysis reveals that all 5 major mesenchymal cell groups have a shared core response as well as subtype-specific responses to pneumococcal pneumonia. (**A**) Heatmap showing unsupervised hierarchical clustering of genes induced at least 2-fold in pneumonia in at least 1 mesenchymal cell subset — fibroblasts (Fibro), myofibroblasts (Myo), smooth muscle cells (SMCs), pericytes (Peri), and mesothelial cells (Meso) — showing both cell-specific and shared core responses in the single-cell RNA-sequencing experiment (3 mice pooled per treatment). Colors along the top of the heatmaps indicate treatment groups: naive (blue), pneumonic (orange), and resolved (black). (**B**) Manual curation of genes revealed a list of 59 genes induced in all 5 mesenchymal cell subtypes during acute pneumonia. (**C**) All 5 cell subtypes also had subset-specific responses based on manual curation. (**D**) Heatmap shows the mean activity of the top 25 transcription factors with variable activity across cell types. (**E**) Reactome pathway enrichment analysis shows pathways potentially involved in the core and cell-specific responses. Pathways with adjusted *P* < 0.2 were selected for visualization.

To study shared mesenchymal cell response to pneumonia, genes that were increased in all 5 mesenchymal cell subtypes were identified via both unsupervised clustering and manual curation. For unsupervised clustering, the top 1,000 genes induced during acute pneumonia were identified for each of the mesenchymal cell types. Genes were then further restricted to only those that increased expression in the pneumonic groups by at least 2-fold in at least 1 cell type. This produced a list of 728 genes, whose expression data were imported into Morpheus (51) for hierarchical clustering to reveal both subtype-specific as well as shared responses (Figure 2A). This analysis revealed groups of genes selectively induced in 1 or several cell types, as well as a cluster of 60 genes that was induced in all 5 cell types. For manual curation, the 1,000 transcripts that most increased in response to infection were identified for each cell type, and those that were shared across lists from all mesenchymal cell types were collated, eliminating all those not increased at least 2-fold, and had an average normalized expression level greater than 0.5 in the pneumonic lung in all 5 cell types. A set of 59 genes was identified as induced in all mesenchymal cell types during acute pneumonia (Figure 2B). Thus, both unsupervised clustering and manual curation analyses suggest that a core set of genes was induced during pneumonia across all mesenchymal cell types in the lung. The gene lists generated by these 2 analyses were overlapping. Twenty-nine genes identified by either analysis included genes encoding cytokines and chemokines (*Cxcl1* and *Cxcl10*), antigen presentation (*H2.T22*, *Psmb9*, *Psmb10*, *Psme1*, and *Psme2*), immune processes (*Nfkbia*, *Phf11d*, *Rtp4*, *Samhd1*, and *Sod2*), apoptosis (*Birc2* and *Xaf1*), interferon-stimulated genes (*Bst2*, *Gbp2*, *Gbp3*, *Ifi35*, *Ifi47*, *Ifit1*, *Ifit3*, *Igtp*, *Iigp1*, *Isg15*, *Irf7*, and *Irgm1*), and others (Supplemental Table 1). We interpret these results to mean that all mesenchymal cells become activated in a pneumonic lung and that a component of these responses is shared by all 5 lung mesenchymal cell types.

To determine if these core responses were specific to mesenchymal cells during pneumococcal pneumonia, the core set of genes was reviewed in transcriptomic data from alveolar macrophages (16) or epithelial cells (52) that we had previously collected from mice with pneumococcal pneumonia. Of the 59 mesenchymal core response genes, *Susd6* was absent from the alveolar macrophage dataset. Alveolar macrophages shared a significant increase in 18 of the 58 core genes analyzed in both datasets but a significant decrease in 3 of the genes (Supplemental Figure 2A). This suggests that the core mesenchymal gene response is not shared with alveolar macrophages. *F830016B08Rik*, *Gbp7*, and *Susd6* were missing from the epithelial cell dataset, leaving 56 genes to analyze. Epithelial cells shared a significant increase in 52 of the 56 core genes analyzed (Supplemental Figure 2B). Epithelial cells showed a significant decrease in *Mif* expression and no change in *Mt1*, *Phf11d*, and *Vmp1*. These data suggest that our core mesenchymal cell response may extend to cells from other lineages as well. To determine whether these core genes can be generalized across pneumonia models, the pneumococcus gene set was compared with a publicly available SARS-CoV-2 dataset from fibroblasts (53). Of the 59 genes upregulated in all mesenchymal cells during *S*. *pneumoniae* infection, only 20 genes were also upregulated in fibroblasts during SARS-CoV-2 infection (Supplemental Figure 2C). Thus, while there appear to be some shared responses, most genes in the core mesenchymal response are specific to pneumococcal pneumonia.

In addition to the shared core response, the unsupervised clustering analysis also suggested cell type–specific responses (Figure 2A). To search for cell type–specific responses using manual curation, the top 350 genes induced during acute infection were restricted to genes increasing at least 2-fold compared with naive cells and with average normalized expression levels greater than 0.5 in the pneumonic group. Genes that met these criteria in only 1 cell type were considered cell type specific. All 5 cell types had specific responses to pneumonia based on the manual curation (Figure 2C). Unsupervised clustering aggregated a set of 47 genes that were induced in mesothelial cells alone; this set included all the genes identified using manual curation, such as *Chil1*, *Anxa8*, and *Prg4* (Supplemental Table 2). The set induced in pericytes alone included 35 genes, such as *Cd40*, *Flt1*, and *Cnn3*, with 12 shared between clustering methods (Supplemental Table 3). A set of 30 transcripts represented fibroblast-specific induction, which included chemokines (*Cxcl5* and *Cxcl13*) and ECM proteins (*Col4a1*, *Col4a2*, *Tnc*, and *Vcan*); 20 were shared between analytic methods (Supplemental Table 4). The unsupervised clustering approach did not reveal prominent myofibroblast-specific responses, but manual curation suggested that these cells induced a set of 7 genes, including *Vimp* and *Tnfaip6* (Supplemental Table 5). Smooth muscle cells selectively increased expression of a cluster of 15 genes, which included the cell adhesion molecule *Vcam1* and the immunoregulatory ligand *Cd200*, 4 of which were shared between both clustering methods (Supplemental Table 6).

Transcription factor activity was inferred from these data using decoupleR. Inferred activities of a set of transcription factors including interferon (IFN) regulatory factors (*Irf1*, *Irf2*, *Irf5*, *Irf7*, and *Irf9*) and 3 nuclear factor κB proteins (*Rela* encoding p65, *Rel* encoding c-Rel, and *Nfkb1* encoding p50 and p105) were strongly increased in all 5 mesenchymal cell types during pneumonia (Figure 2D). This is suggestive of a pan-mesenchymal innate immune response during pneumococcal infection, regardless of cell type or location. Fibroblasts exhibited increased activity for *Snai2* and *Tcf711* in response to infection. Other transcription factor activities that were elevated in specific cell types without regard to infection included *Foxl2* and *Nfe2l3* in smooth muscle cells, *Ebf1* and *Glis3* in pericytes, and *Znf24* in mesothelial cells. These data suggest that mesenchymal cells have both pan-mesenchyme and subset-specific transcription factor activities.

To infer biological processes from these transcriptome changes, Reactome pathway enrichment analysis was performed on both the core and subset-specific gene lists (Figure 2E). The set of core mesenchymal genes mediate broad immune signaling pathways, such as antigen presentation and cytokine signaling. The genes modified in fibroblasts were involved in ECM dynamics, such as collagen and laminin generation, assembly, and interactions. Pericytes were tied most strongly to growth hormone signaling. Additional pathway associations were weakly implicated. These data suggest shared immune activities across mesenchymal cell types, as well as some subset-specific biological responses during pneumococcal pneumonia.

Autocrine and paracrine communications among the mesenchymal cells were identified by CellChat analyses (Supplemental Figure 3). All outgoing signals from pericytes, smooth muscle cells, and myofibroblasts exhibited an increase in interactions during pneumonia compared with naive cells (Supplemental Figure 3A). Fibroblast interactions with pericytes and mesothelial cells diminished, but their interactions with smooth muscle cells and myofibroblasts increased. Interestingly, while numbers of interactions with other cell types tended to increase in response to pneumonia, the strength of those signals tended to decrease when compared with naive cells for most mesenchymal cell types (Supplemental Figure 3B). Among pneumonic mesenchymal cells, pericytes had the most outgoing signals, followed by fibroblasts; mesothelial cells, pericytes, and smooth muscle cells had similar levels of incoming signals (Supplemental Figure 3C). Collagen was the most common outgoing and incoming signal of all mesenchymal cells, followed by *CypA*, which encodes cyclophilin A.

### Fibroblasts are heterogeneous in the resting lung, including alveolar and adventitial subsets.

**Figure 3:**
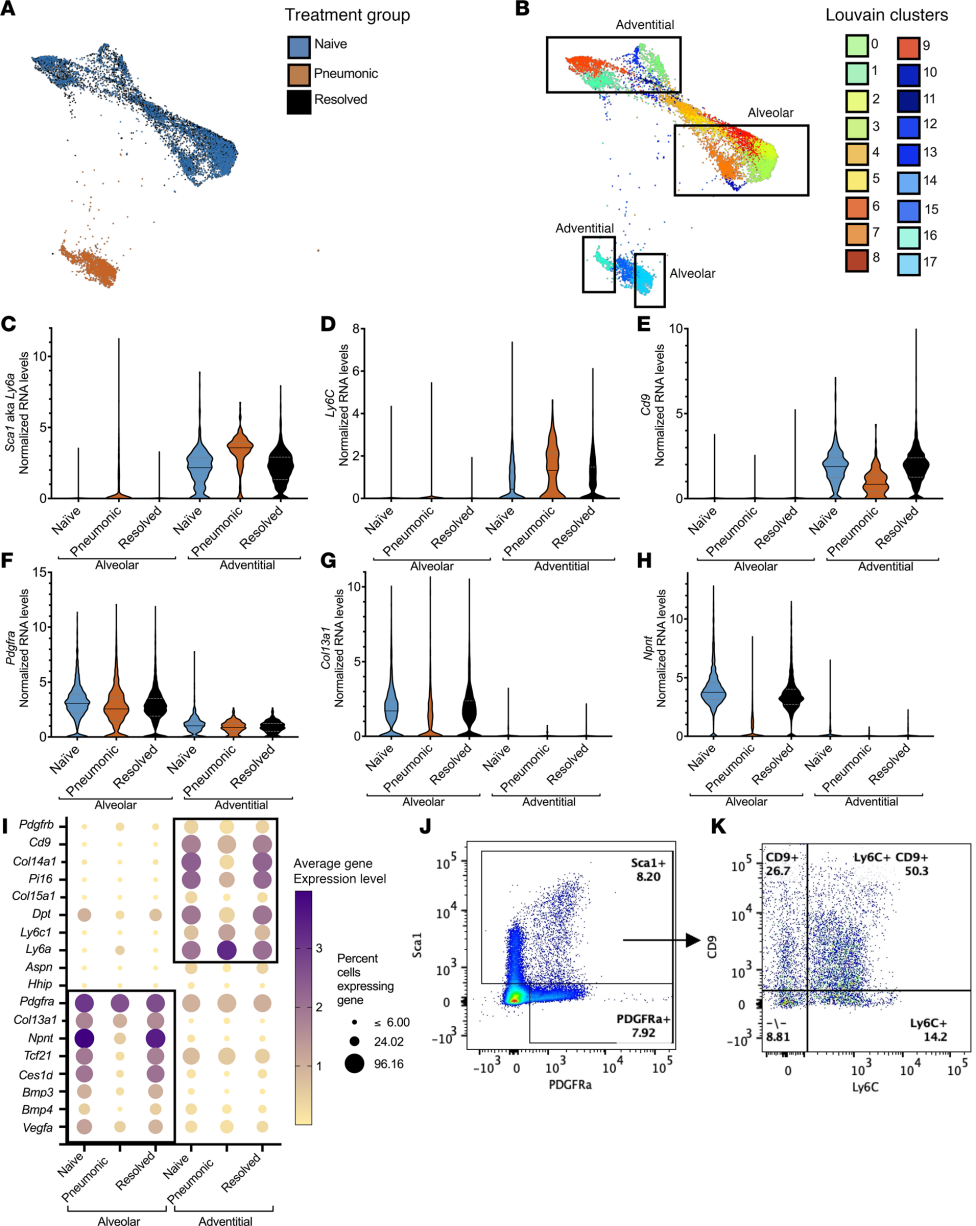
Fibroblasts are heterogeneous in the resting lung but ultimately group into alveolar and adventitial fibroblast subsets. (**A**) SPRING plots show that fibroblasts isolated from naive (blue) and resolved (orange) mice overlap whereas fibroblasts from pneumonic (black mice were nonoverlapping with the other treatment groups in the single-cell RNA-sequencing experiment (3 mice pooled per treatment). (**B**) SPRING plots show heterogeneity of lung fibroblasts with 17 distinct Louvain clusters. (**C**–**E**) Violin plots illustrate that fibroblasts can be classified as adventitial based on the expression of *Sca1*, aka *Ly6a*; *Ly6C*; and *Cd9*. Violin plots show the mean ± the range. (**F**–**H**) Violin plots illustrate that fibroblasts can be classified as alveolar based on the expression of *Pdgfra*, *Col13a1*, and *Npnt*. (**I**) A correlation dot plot further exemplifies that fibroblasts have distinct alveolar or adventitial fibroblast gene signatures. (**J**) Flow cytometry dot plots verify that CD45^–^CD31^–^CD321^–^ mesenchymal cells express Sca1 (encoded by *Ly6a*) or PDGFRα proteins on their surface. (**K**) Further gating on Sca1^+^ cells shows that over 60% cells are Ly6C^+^ or CD9^+^. Dot plots are representative of 2 separate experiments (*n* = 10).

Fibroblasts were the most abundant mesenchymal cell type in the lungs, at least 5-fold more numerous than any of the other cell types in every group of mice. To understand these cells better, they were separately reclustered and analyzed. The fibroblasts from naive and recovered lungs again overlapped, whereas fibroblasts from pneumonic lungs clustered into a different and nonoverlapping population (Figure 3A). Louvain clustering identified 18 subsets of fibroblasts, 15 within naive and recovered lungs and 3 within pneumonic lungs (Figure 3B). The 15 naive and resolved clusters aggregated in one large butterfly-shaped cluster. Using genes that differentiate subsets recognized as adventitial or alveolar fibroblasts (30, 33, 54, 55), as well as Louvain clustering of this dataset, this large butterfly cluster distinguishes adventitial *Sca1^+^Ly6C^+^Cd9^+^* cells (clusters 0, 1, 6, 12, and 16; Figure 3, C–E) and alveolar *Pdgfra^+^Col13a1^+^Npnt^+^* (clusters 2, 3, 7, 8, 9, 10, and 17; Figure 3, F–H). Louvain clusters 4, 5, 11, and 13 in naive and resolved groups along with cluster 15 in the pneumonic group exhibited expression of both adventitial and alveolar fibroblast genes and thus may be transitional. In addition to being *Pdgfra^+^Col13a1^+^Npnt^+^*, the fibroblasts in the right-side clusters also expressed *Ces1d* and *Tcf21* (Figure 3I and Supplemental Figure 4, A and B), which mark alveolar fibroblasts specifically (56). Consistent with their potential alveolar localization and function, these cells expressed *Bmp3*, *Bmp4*, and *Vegfa* (Figure 3I and Supplemental Figure 4, C–E), mesenchymal products essential to supporting alveolar epithelial cells and pulmonary capillary endothelial cells (34). Going forward, cells in the clusters to the right will be referred to as alveolar fibroblasts.

The *Sca1^+^Ly6C^+^Cd9^+^* cells expressed *Pi16*, *Dpt*, and *Col14a1* (Figure 3I and Supplemental Figure 4, F–H), suggesting they are the adventitial fibroblasts found across multiple organs (56). *Col15a1* has been used to distinguish a subset of adventitial fibroblasts, but this gene was not as strongly expressed as *Pi16* in lung fibroblasts (Figure 3I and Supplemental Figure 4, E and J). Flow cytometry analysis of CD45^–^CD325^–^CD31^–^ cells showed distinct PDGFRα^+^ single-positive, Sca1^+^ single-positive, and Sca1^+^PDGFRα^+^ double-positive populations (Figure 3J). Of the Sca1^+^ cells, over 60% were Ly6c^+^ (Figure 3K), suggestive of an adventitial fibroblast population (30). Previous reports have shown that PDGFRα, Sca1, and CD9 can be used to distinguish alveolar, adventitial, and peribronchial fibroblasts (56). Here we show that over 80% of the Sca1^+^ cells were also CD9^+^ (Figure 3K), suggesting that CD9 is not a distinctive marker for fibroblast subsets. Therefore, going forward, cells in the left clusters will be referred to as adventitial fibroblasts.

Previous reports identify cells as peribronchial fibroblasts based on expression of *Aspn*, *Hhip*, *Fgf18*, *Cthrc1*, and *Wif1*, so we looked for such cells in our dataset (56). All these genes were most highly expressed in a distinct population of cells that clustered away from matrix fibroblasts (clusters 14, 17, and 21 in Figure 1B). This set of cells expressed *Acta2* and *Mustn1* in addition to *Aspn* and *Hhip* (Figure 1C), so we identified them as myofibroblasts (33, 39, 41, 42). Regardless of nomenclature, the transcriptomic analyses reveal that these are a distinct smaller subset of cells that is very different from the matrix fibroblasts, which are more abundant in the lung, including both *Pdgfra^+^Col13a1^+^Npnt^+^Ces1d^+^* alveolar fibroblasts and *Sca1^+^Ly6C^+^Cd9^+^Pi16^+^Col14a1^+^* adventitial fibroblasts.

**Figure 4:**
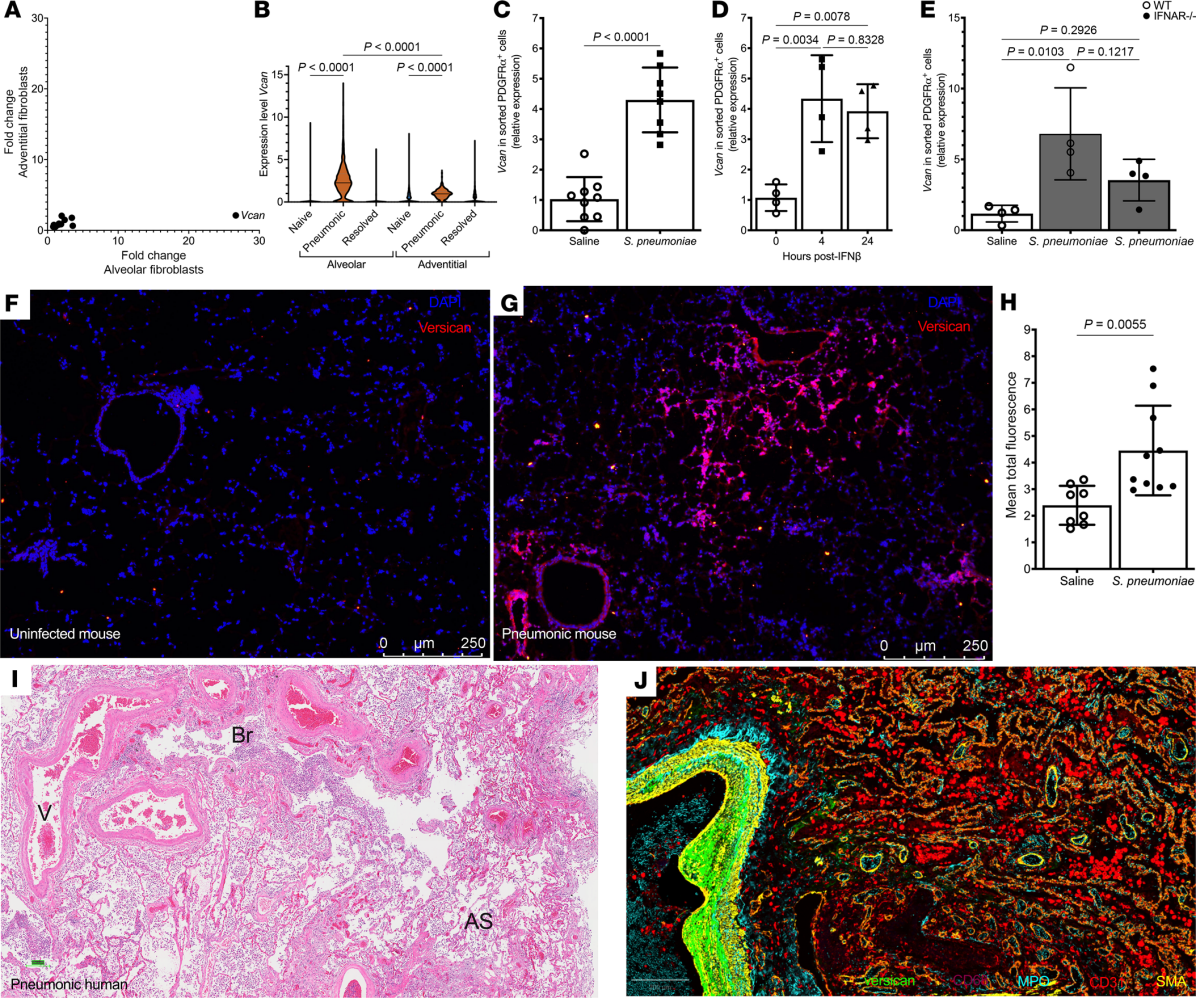
Fibroblasts increase versican expression and production in response to type I interferons and pneumonia. (**A**) Alveolar fibroblasts have a 27-fold induction in versican expression during pneumonia, compared with 2-fold induction in adventitial fibroblasts. (**B**) Violin plots from single-cell RNA-sequencing experiment (3 mice pooled per treatment) show that alveolar fibroblasts from pneumonic mice have significantly higher *Vcan* expression compared with adventitial fibroblasts. Significance was determined by 1-way ANOVA followed by Tukey’s multiple comparisons test. (**C**) qRT-PCR analysis. Sorted PDGFRα*^+^* fibroblasts from mice 24 hours after i.t. *S*. *pneumoniae* (*n* = 8) have higher expression of *Vcan* than those from saline-treated (*n* = 7) mice. *Ifnar^–/–^* mice are designated with filled circles. Mice infected with *S*. *pneumoniae* are designated by dark bars. Significance was determined by unpaired *t* test. *Ifnar*^–/–^, IFN-ɑ receptor knockout; *S*. *pneumoniae*, *Streptococcus pneumoniae*. (**D**) *Vcan* is expressed in sorted PDGFRα*^+^* fibroblasts from mice 4 (*n* = 4) and 24 (*n* = 4) hours after i.t. recombinant IFN-β. Significance was determined by 1-way ANOVA followed by Tukey’s multiple comparisons test. (**E**) *Vcan* is expressed in PDGFRα*^+^* fibroblasts isolated from *Ifnar^–/–^* mice 24 hours (*n* = 4) after *S*. *pneumoniae*. Significance was determined by 1-way ANOVA followed by Tukey’s multiple comparisons test on log-transformed data. Representative immunofluorescence micrographs from 2 experiments show versican (red) staining is minimal in saline-treated (**F**, *n* = 8) mice but more prominent in lungs of mice 24 hours after *S*. *pneumoniae* (**G**, *n* = 10); DAPI-labeled cell nuclei in blue. (**H**) This was verified with quantification. Significance was determined by unpaired *t* test. (**I**) H&E and (**J**) multiplex immunofluorescence micrographs of the same lung biopsy from a human with pneumonia show that versican (green) is present around larger blood vessels (V), bronchioli (Br), and alveolar septae (AS); myeloperoxidase-labeled (cyan) neutrophils, CD68-labeled (magenta) macrophages, CD31-labeled (red) endothelial cells, smooth muscle actin–labeled (yellow) SMCs, and DAPI-labeled (gray) cell nuclei. **I** and **J** were taken at the same magnification, and the scale bar in **J** indicates 200 µm. All data are mean ± SD; data points are values from individual mice (**C**–**E**, and **H**).

The main function of matrix fibroblasts is to produce and maintain ECM proteins to support tissue architecture and cell-to-cell interactions. Therefore, we analyzed transcripts for ECM proteins in alveolar and adventitial fibroblasts (Supplemental Figure 5). Some transcripts showed differential expression at baseline (e.g., more decorin transcripts in the adventitial fibroblasts and more fibronectin transcripts in the alveolar fibroblasts). Of the matrix genes examined, after meeting our >2-fold induction and >0.5 normalized gene expression inclusion criteria, only *Vcan* was induced during pneumonia (Figure 4A). It was induced in both cell types but showed stronger induction (27-fold vs. 2-fold, Supplemental Figure 5) and greater expression levels in alveolar fibroblasts compared with adventitial fibroblasts in the infected lung (Figure 4B). Since versican (encoded by *Vcan*) can play roles in leukocyte recruitment (57–59), we examined this in the context of pneumonia.

### Versican is induced in fibroblasts and accumulates in infected lungs.

Versican is a large chondroitin sulfate protein with complex regulation and many functions in inflammatory infections and diseases (59, 60). To verify that pneumococcal infection induced versican expression in alveolar fibroblasts, mice were administered either sterile saline or *S*. *pneumoniae*, and PDGFRα^+^ mesenchymal cells were isolated 24 hours later. RNA was harvested from the sorted cells, and quantitative reverse transcriptase PCR (qRT-PCR) was performed to examine versican expression. There was significantly more *Vcan* mRNA in the PDGFRα^+^ fibroblasts from lungs with pneumococcal infection, compared with those from uninfected lungs (Figure 4C). These data support the postulate that pneumonia induces versican expression in alveolar fibroblasts.

Type I IFNs can stimulate versican production (57, 61). To determine whether this applies to alveolar fibroblasts in the lung, recombinant murine IFN-β was intratracheally (i.t.) instilled, and PDGFRα^+^ fibroblasts were sorted for qRT-PCR analyses. *Vcan* expression increased by 4 hours after IFN-β treatment, which persisted through at least 24 hours (Figure 4D). To test whether *Vcan* expression was dependent on type I IFNs, wild-type and *Ifnar1*^–/–^ mice were infected with pneumococcus. PDGFRα^+^ cells were isolated after 24 hours, and RNA was harvested for qRT-PCR. *Vcan* expression was significantly increased in PDGFRα^+^ cells from pneumonic lungs of both wild-type and *Ifnar1*^–/–^ mice compared with cells from uninfected lungs (Figure 4E). There was a trend toward decreased expression in the *Ifnar1*^–/–^ mice with infections (Figure 4E), suggesting that type I IFNs may aid in versican expression. We conclude that type I IFN signaling stimulates versican expression in alveolar fibroblasts but is not required for the induction observed during pneumococcal pneumonia.

To visualize versican in mouse lungs, we administered wild-type mice either sterile saline or pneumococcus i.t. After 24 hours, left lobes were harvested and versican immunofluorescence staining was performed. In the saline group, versican was sparse and localized near blood vessels and major airways (Figure 4F). In the pneumonic lungs, versican was more abundant and more diffusely distributed, including alveolar staining that was not observed in the uninfected lung (Figure 4G). Quantifying the fluorescent signal for versican revealed a significant increase in the infected compared with the uninfected lungs (Figure 4H). To evaluate versican production in humans, serial sections from people who died with autopsy-diagnosed pneumonia were stained for histopathology examination and versican expression. Regions with pronounced suppuration (Figure 4I) showed prominent versican staining (Figure 4J). Versican localized to expanded matrix material within perivascular cuffs flanking dense neutrophil aggregates as well as adjacent alveolar interstitium. Thus, versican accumulates in alveolar regions of infected lungs in spatial proximity to leukocyte influx.

### Versican from Pdgfra-expressing cells is dispensable for acute responses to S. pneumoniae infection.

**Figure 5:**
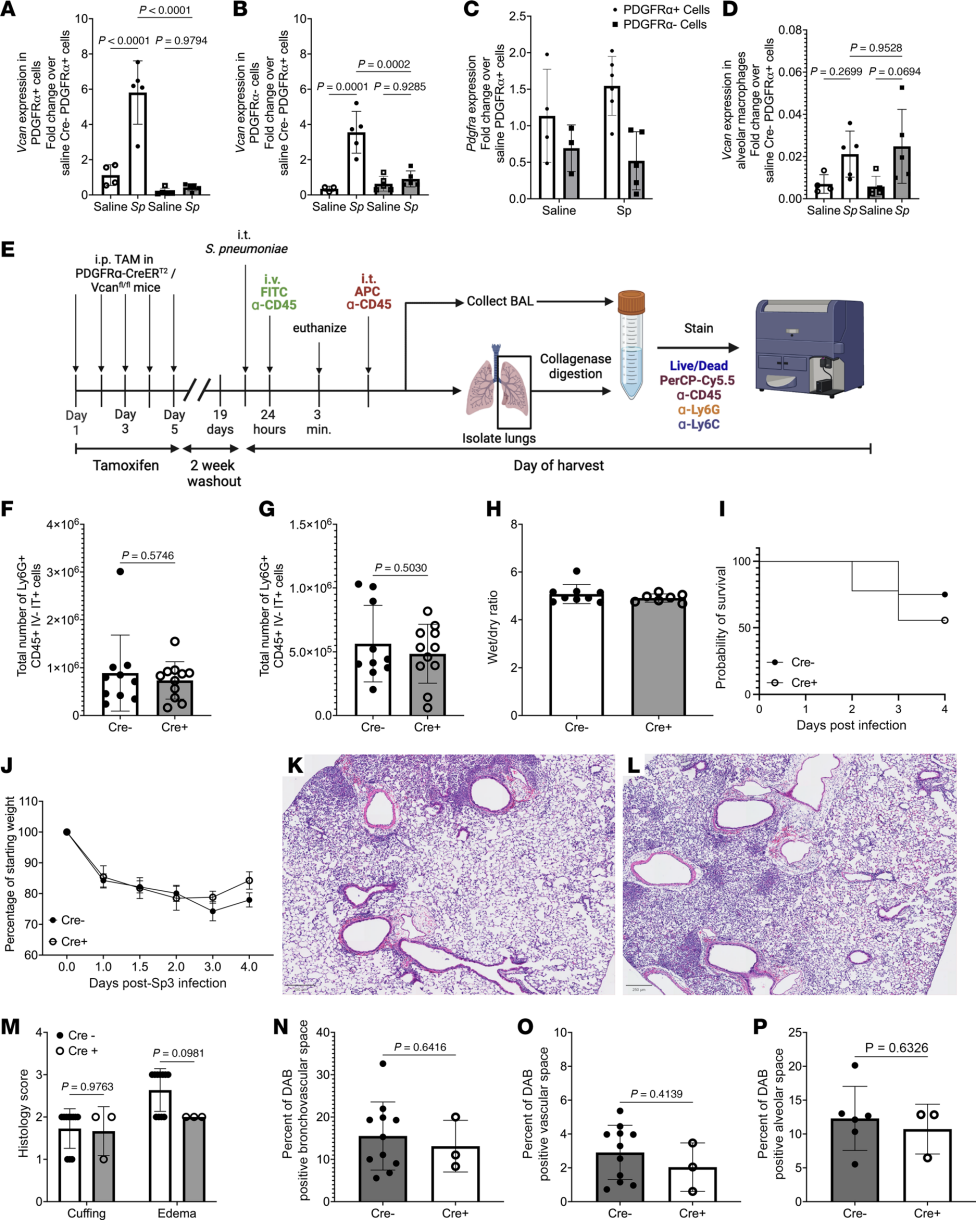
PDGFRα^+^ cell–derived versican does not play a role in inflammation during pneumonia. *Vcan* expression is ablated in sorted (**A**) PDGFRα^+^ and (**B**) PDGFRα^–^ mesenchymal cells from *Pdgfra*^Cre–ERT2+^ *Vcan*^tm1.11Cwf^ (Cre^+^) but not *Pdgfra*^Cre–null^ *Vcan*^tm1.11Cwf^ (Cre^–^) mice. (**C**) qRT-PCR analysis of sorted PDGFRα^+^ and PDGFRα^–^ mesenchymal cells shows that *Pdgfra* is expressed in cells that do not express surface PDGFRα. (**D**) *Vcan* expression is unchanged in sorted alveolar macrophages. Significance was determined by 1-way ANOVA followed by Tukey’s multiple comparisons test (*n* = 4–5 per treatment group per genotype). (**E**) Schematic shows the timeline of neutrophil compartmentalization study 24 hours after i.t. *S*. *pneumoniae* (created using BioRender). Flow cytometry analysis revealed that (**F**) airspace and (**G**) interstitial neutrophil number were not different in Cre^–^ (*n* = 11) and Cre^+^ (*n* = 10) mice. BAL, bronchoalveolar lavage. (**H**) Edema was not significantly different in lungs of Cre^–^ (*n* = 9) or Cre^+^ (*n* = 7) mice 24 hours after *S*. *pneumoniae*. (**I**) Survival and (**J**) weight loss after lethal dose of *S*. *pneumoniae* and subsequent antibiotic treatment showed that Cre^–^ (*n* = 5) and Cre^+^ (*n* = 8) mice have similar responses to pneumococcal infection. Similarly, H&E staining of lung sections showed no remarkable difference in inflammation or cell infiltration in (**K**, *n* = 6) Cre^–^ and (**L**, *n* = 5) Cre^+^ mice. Images representative from 2 separate experiments. Scale bars represent 250 μm. (**M**) Histological analysis showed no difference in perivascular cuffing or edema. Significance was determined by 2-way ANOVA followed by Holm-Šidák multiple comparisons test. Quantification of versican IHC staining showed no remarkable difference in location of versican in the bronchovascular (**N**), vascular (**O**), or alveolar (**P**) regions. Significance was determined by unpaired *t* test. All data are mean ± SD; data points are values from individual mice (**A**–**D**, **F**–**H**, and **M**–**P**) or represent the mean (**J**).

To test how alveolar fibroblast-derived versican influences immune and physiological processes during pneumonia, we crossed the *Pdgfra*^CreERT2^ transgene into *Vcan*-floxed mice. Tamoxifen was administered to Cre-negative (Cre^–^) and Cre-positive (Cre^+^) littermates, and *Vcan* expression was successfully abrogated in Cre^+^ mice (Figure 5A). *Vcan* induction was also abrogated in PDGFRα^–^ mesenchymal cells (Figure 5B). Our scRNA-Seq data suggested that *Pdgfra* mRNA was widely expressed by lung fibroblasts, albeit more so in the alveolar fibroblasts (Figure 3F). Consistent with this, *Pdgfra* mRNA was detectable in both sets of sorted mesenchymal cells (Figure 5C). Thus, *Pdgfra*-mediated gene targeting extends beyond the alveolar fibroblasts to include other mesenchymal cells. Alveolar macrophages are additional sources of versican in the lung (61–63), and these cells do not express PDGFRα. In contrast with the mesenchymal cell populations examined, the induction of versican in alveolar macrophages was unaffected by the *Pdgfra*-driven Cre transgene (Figure 5D). We conclude that tamoxifen treatment of Cre^+^ mice effectively targets *Pdgfra*-expressing cells, including alveolar fibroblasts plus other mesenchymal cells. To examine the roles of versican in neutrophil recruitment and localization during pneumonia, we designed an experiment to stain and analyze CD45^+^ cells in the blood, alveolar space, and interstitium (Figure 5E). Mice received an FITC-labeled antibody against CD45 intravenously to label all intravascular leukocytes. An APC-labeled antibody against CD45 was also delivered i.t. to label airspace leukocytes. BAL and left lung lobes were collected to be analyzed using flow cytometry (Supplemental Figure 6). The numbers of neutrophils in the airspace and interstitium of pneumonic lungs were not significantly affected by *Vcan* ablation in *Pdgfra*-expressing cells (Figure 5, F and G). Of the extravascular neutrophils, most were in the airspace, and this also was unaffected by *Vcan* ablation in *Pdgfra*-expressing cells. The accumulation of pulmonary edema was also unaffected by *Vcan* mutation at this time point (Figure 5H). We considered a longer time frame, to determine whether versican expression by *Pdgfra*-expressing cells might influence later stages of pneumonia. Because the bacterial infection is typically lethal within 3 days, mice were treated with antibiotics beginning 36 hours after infection. In this setting, both Cre^–^ and Cre^+^ mice had about a 60% survival through 4 days (Figure 5I). Surviving mice were regaining weight (Figure 5J) and had histopathology findings consistent with resolving pneumonia (Figure 5K). Versican targeting in *Pdgfra*-expressing cells did not affect these outcomes (Figure 5, I–M). Histopathological analysis of lung sections from Cre^–^ and Cre^+^ samples revealed no significant differences in perivascular, bronchovascular, or alveolar inflammation and injury (Figure 5M and Supplemental Figure 7, A and B). Immunohistochemical staining of versican revealed no significant differences in versican localization in the lungs (Figure 5, N–P, and Supplemental Figure 7, C and D). These data suggest that induction of versican expression in *Pdgfra*-expressing cells, including alveolar fibroblasts, is not essential for neutrophil recruitment or injury during pneumonia or for the ensuing recovery.

### Alveolar fibroblasts respond more strongly than adventitial fibroblasts.

**Figure 6:**
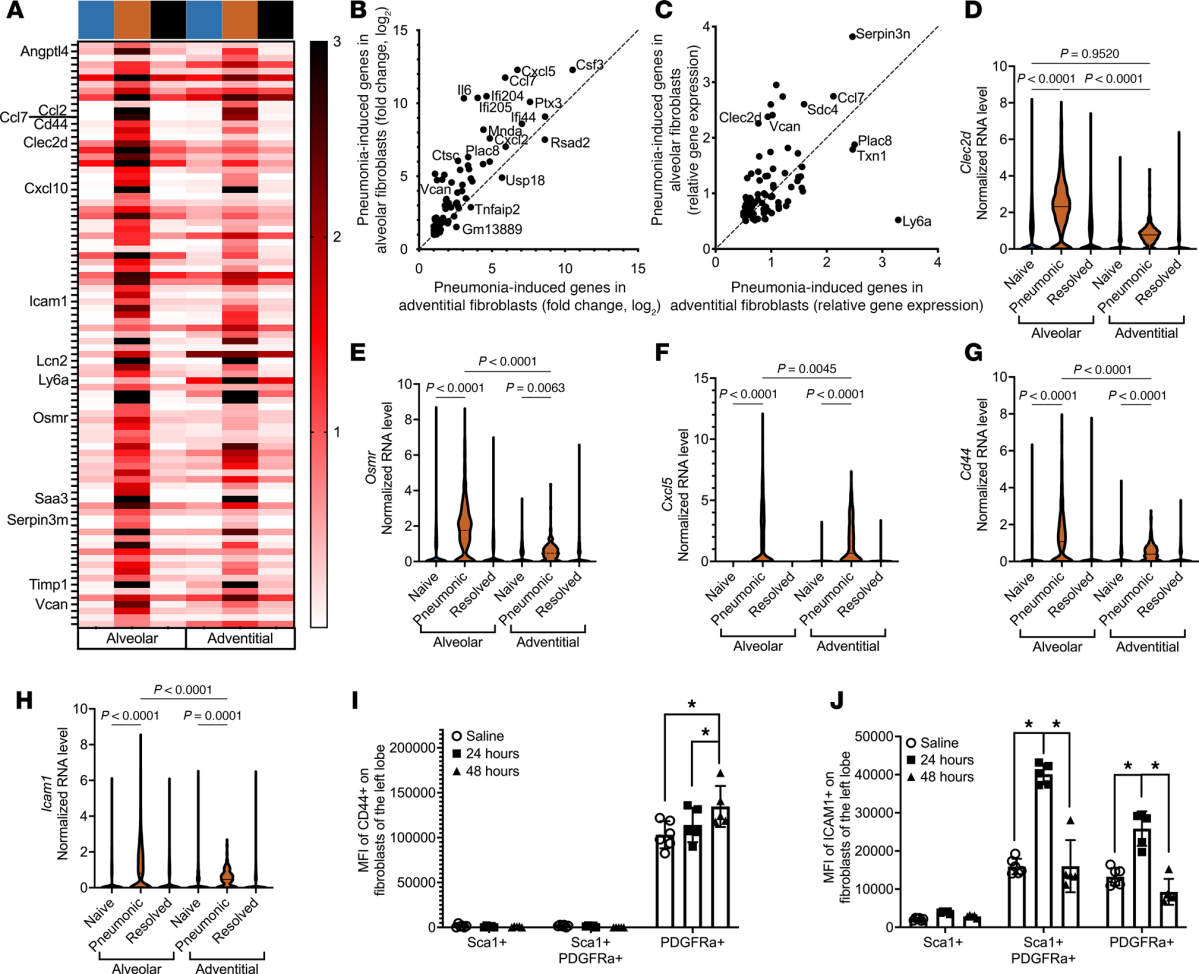
Alveolar fibroblasts are more responsive to pneumococcal pneumonia. (**A**) Heatmap representation shows gene expression of core matrix fibroblast response genes induced during 24 hours of *S*. *pneumoniae* infection. Colors along the top of the heatmaps indicate treatment groups: naive (blue), pneumonic (orange), and resolved (black). XY scatterplot representation of the (**B**) log_2_(fold-change) and (**C**) average gene expression of core matrix fibroblast genes, illustrating that alveolar fibroblasts respond more robustly than adventitial fibroblasts. Violin plots show that alveolar fibroblasts have stronger expression of (**D**) *Clec2d*, (**E**) *Osmr*, (**F**) *Cxcl5*, (**G**) *Cd44*, and (**H**) *Icam1*, genes associated with leukocyte recruitment and migration. Significance was determined by 1-way ANOVA followed by Tukey’s multiple comparisons test. Flow cytometry analysis of mesenchymal cells shows that PDGFRα*^+^* single-positive cells express surface (**I**) CD44 and (**J**) ICAM1 in both naive and pneumonic mice, whereas Sca1^+^ single-positive cells do not express either protein, even during pneumonia (*n* = 5–6 mice per time point). A PDGFRα^+^Sca1^+^ double-positive population showed a distinct phenotype and were negative for CD44 but positive for ICAM1. Significance was determined by 2-way ANOVA followed by Tukey’s multiple comparisons test (*P < 0.05). All data are mean ± SD; data points are values from individual mice (**I** and **J**).

Since we noted versican to be more strongly induced in alveolar fibroblasts compared with adventitial fibroblasts during pneumonia, we considered whether gene induction patterns may distinguish these fibroblast subsets. We identified gene sets induced in alveolar fibroblasts and gene sets induced in adventitial fibroblasts by collating the 1,000 genes most strongly induced in each subset and eliminating any genes that increased less than 2-fold, had normalized expression less than 0.5 during pneumonia, or were in the core mesenchymal response dataset (Figure 2A). This returned the same set of 80 genes from each of the 2 fibroblast subsets. Thus, a shared set of genes is induced during pneumonia in both adventitial and alveolar fibroblasts (Figure 6A). Unlike cell type–specific responses to pneumonia that distinguish each of the 5 major mesenchymal cell clusters (Figure 2), no genes induced by pneumonia distinguished the alveolar versus adventitial fibroblasts. While the same genes were induced in both fibroblast subsets during pneumonia, the alveolar fibroblasts tended to respond more robustly, whether plotted as fold-induction due to pneumonia (Figure 6B) or as average mRNA levels during pneumonia (Figure 6C). The shared gene list included multiple immune-related genes associated with cell death, migration, and adhesion, such as *Clec2d*, *Osmr*, *Cxcl5*, *Cd44*, and *Icam1*, all of which were induced to a significantly greater extent in the alveolar fibroblasts (Figure 6, D–H). CD44 and ICAM1 expression was analyzed at the protein level using flow cytometry in independent sets of experiments. Two dominant populations of mesenchymal cells had either PDGFRα^+^Sca1^–^ or PDGFRα^–^Sca1^+^ surface markers, interpreted as alveolar and adventitial fibroblasts, respectively. There was also a third smaller population that was double positive for Sca1 and PDGFRα. CD44 protein was observed only on PDGFRα^+^Sca1^–^ mesenchymal cells, and it increased during pneumonia (Figure 6I). ICAM1 was expressed by all PDGFRα*^+^* mesenchymal cells and induced during pneumonia (Figure 6J). The fact that the PDGFRα^+^Sca1^+^ cells did not consistently match to either alveolar PDGFRα^+^Sca1^–^ mesenchymal cells or adventitial PDGFRα^–^Sca1^+^ mesenchymal cells across these 2 signals suggests that this population may be a distinct mesenchymal cell type. Combined with the inability to define some of the matrix fibroblasts to either the adventitial or alveolar subsets of fibroblasts (clusters 4, 5, 11, 13, and 15 in Figure 3B), these flow cytometry data further support additional levels of heterogeneity among mouse lung fibroblasts. The fact that both CD44 and ICAM1 proteins increase during acute pneumonia on alveolar but not adventitial fibroblasts supports the conclusion that alveolar fibroblasts respond more strongly than adventitial fibroblasts during pneumococcal pneumonia.

## Discussion

While early studies suggested that mesenchymal cells have an immune role during lung infection (29), little is known about whether and how the various mesenchymal cell subsets have distinct and specific responses to infection. scRNA-Seq revealed 5 mesenchymal cell types in the lungs, regardless of treatment: fibroblasts, myofibroblasts, smooth muscle cells, pericytes, and mesothelial cells. Of these 5 subsets, fibroblasts made up over 70% of mesenchymal cells collected, similar to other reports (30, 33, 64). When clusters were broken up based on their treatment group, all mesenchymal cells responded to pneumonia and clustered away from naive cells. These data suggest that all mesenchymal cells respond and help mediate the integrated immune activities in this infected tissue (21, 64, 65). After responding to pneumonia, the lung mesenchymal cell pool reverts to the naive state. This conclusion is based on SPRING plots from the naive and resolved lungs, which overlap to suggest no new mesenchymal cell types and no substantial alteration to the resting transcriptomes of the cell types present. This contrasts with what has been observed with leukocytes. After pneumonia recovery, the lungs contain new lymphocytes, including resident memory T cells and B cells (10, 12, 66–68). Also, alveolar macrophages exhibit “trained immunity,” where cells from the recovered lung have different resting transcriptomes compared with those in naive lungs (15, 16, 69–71). Mesenchymal cells do not appear to contribute to the remodeled immunity of recovered lungs like leukocytes.

A common set of genes increased in all 5 mesenchymal cell groups during acute pneumonia, suggesting a core response conserved across cell types. Some genes in this core response encode chemokines, such as *Cxcl1* and *Cxcl10*. These data agree with precedent data showing that pericytes induce expression of CXCL1 and CXCL10 in response to IL-17 stimulation (24). These chemokines and other core response genes of mesenchymal cells may influence leukocyte recruitment, migration, and activation in the interstitial space.

To determine if the core response was specific to mesenchymal cells, the list of 59 core genes was compared with transcriptomic responses during pneumonia in alveolar macrophages (16) and epithelial cells (52). When compared with alveolar macrophages, only a handful of the mesenchymal core response genes were upregulated in both cell types, suggesting that the immune cells and mesenchymal cells have distinct responses to pneumococcal pneumonia. When compared with epithelial cells, almost all the mesenchymal core response genes were upregulated in both cell types, suggesting that our mesenchymal core response may be a core structural cell response to pneumococcal pneumonia. These trends remained consistent (data not shown) even if we expanded the core mesenchymal dataset by relaxing the inclusion threshold, suggesting robustness of these mesenchymal cell response distinctions (from alveolar macrophages) and similarities (with epithelial cells). Additionally, to evaluate if the core response was upregulated in other pneumonias, like SARS-CoV-2, the core response genes were compared to publicly available datasets (53). Only 20 genes were shared between mesenchymal cells during pneumococcal infection and fibroblasts during SARS-CoV-2 infection. The genes shared between both infection models are broad immune response genes and are involved in processes such as antigen presentation and cytokine signaling. We conclude that the core mesenchymal response genes reported here are conserved across mesenchymal cells in response to pneumococcus infection, but this should not be generalized to all pneumonias.

Conversely, each mesenchymal cell subset induced some unique genes during pneumonia. Myofibroblasts had the least specific response and only increased expression of 7 specific genes, which included *Vimp* (72, 73) and *Tnfaip6* (74), and therefore suggests antiinflammatory and pro-resolution roles for these cells. Mesothelial cells increased expression of 8 specific genes, including *Chil1*, *Anxa8*, and *Prg4*, all of which influence immunity and inflammation (75–78). By modulating immunity in the pleural space, mesothelial cells may contribute to pneumococcal pneumonia’s propensity to cause empyema. Smooth muscle cells increased expression of 10 specific genes, including *Vcam1* and *Cd200*. Both encode plasma membrane proteins that are ligands for receptors exclusively expressed by leukocytes, CD200R and CD18. These findings highlight the potential paracrine immunomodulatory activity for smooth muscle cells. Pericytes induced expression of 15 specific genes, including *Cd40*, *Flt1*, and *Cnn3*. CD40 is a membrane protein that functions specifically to activate T cells. *Flt1* and *Cnn3* are smooth muscle cell markers and are required for smooth muscle function. These data suggest that pericytes may undergo differentiation into smooth muscle cells during infection in the lungs and agree with reports illustrating that pericytes are progenitors for smooth muscle cells in coronary arteries (79). Fibroblasts increased expression of 22 genes that were not induced in other mesenchymal cells, including *Cxcl5*, *Cxcl13*, *Col4a1*, *Col4a2*, and *Vcan*. CXCL5 and CXCL13 are chemokines for neutrophils and B cells, suggesting that fibroblasts may help position these leukocytes appropriately within connective tissue. Epithelial cells, endothelial cells, and leukocytes can also produce CXCL5 and CXCL13 during infection (11, 80, 81), so these chemokines may be important in a microenvironmental niche. *Col4a1* and *Col4a2* are of interest because they are ECM proteins, consistent with the best recognized role of fibroblasts as matrix synthesizers. Since type IV collagens can dictate levels of CCL5 and CCL7 expression (82), these fibroblast-derived matrix proteins may have immune roles during respiratory infection.

Among ECM proteins, versican is a chondroitin sulfate proteoglycan with diverse roles, including cell differentiation, migration, adhesion, and proliferation, as well as in tissue stabilization and inflammation (58, 83). The chondroitin sulfate is abundantly expressed during lung development, but expression is very low in lungs of healthy adult mice and humans (58, 84, 85). The robust induction of versican, especially in alveolar fibroblasts, led us to suspect it might be involved in neutrophil recruitment. Neutrophil recruitment occurs in alveolar septae during pneumococcal pneumonia (86, 87), and versican has been implicated in neutrophil recruitment (57, 59). scRNA-Seq, qRT-PCR, and immunofluorescence verified that versican is increased during pneumococcal pneumonia both in perivascular and in alveolar interstitial compartments. It is similarly induced in models of Gram-negative bacteria lung infection (62), acute lung injury (88), asthma (89), pulmonary fibrosis (90), influenza (20), and cancer (91). It can act as a scaffold that binds and presents chemokines in haptotactic gradients to leukocytes (92). In the interstitium, it can render the provisional matrix more open and permissive for cell migration. In models where versican is significantly increased, infiltrating leukocytes strongly colocalize with the proteoglycan (93, 94). In the lungs, poly-IC treatment has also resulted in a significant increase in versican, and global knockdown of versican resulted in a significant decrease in leukocytes in the airspaces (63). This is in contrast with our study. While versican is strongly induced in alveolar fibroblasts, its production by *Pdgfra*-expressing cells is dispensable for neutrophil recruitment, pulmonary edema, and resolution after pneumococcal pneumonia. It is possible that versican is not essential for these processes or that versican expression by other cells compensates for the targeting of the versican gene in PDGFRα^+^ cells.

Immunohistochemical staining revealed no remarkable differences in versican protein in infected lungs of mice in which the *Vcan* gene was successfully targeted in *Pdgfra*-expressing mesenchymal cells. While we have demonstrated induction of the *Vcan* gene at the mRNA level, we do not have direct evidence that fibroblasts produce versican protein in response to pneumococcus infection. It is possible that fibroblasts are not secreting versican during pneumococcal pneumonia. However, we also note that other cells are important sources of versican, and these may compensate for the loss of PDGFRɑ^+^ cell–derived versican. Future studies should focus on the cellular sources of the glycoprotein at the protein level. Previous studies have shown that versican can function as a pro-inflammatory or an antiinflammatory molecule, depending on the cells that produce it. Versican produced by macrophages and epithelial cells limits acute pulmonary inflammation (61, 62, 95); however, when produced by fibroblasts, the protein supports a pro-inflammatory microenvironment (59). Future studies will need to determine if macrophage-derived versican can function as a pro-inflammatory molecule when other methods of versican production are altered. One last explanation may be that other proteoglycans compensate for the lack of versican. Other proteoglycans of the lung ECM are syndecan, perlecan, biglycan, decorin, and lumican (58, 84). We focused our attention on versican because it was the only ECM protein significantly induced in response to pneumonia (Figure 4, A and B). However, adventitial fibroblasts had very strong expression of *Dcn*, the gene encoding decorin, in all treatment groups tested (data not shown). Previous reports have shown that high decorin levels correlate with higher leukocyte numbers in the serum of acute coronary syndrome (96). Future studies can examine other proteoglycan levels in these knockout mice and investigate how they may influence the response to pneumonia.

While all mesenchymal cells responded to pneumonia, matrix fibroblasts had very distinct and specific responses to infection. As expected with many transcriptomic studies, this study showed a disconnect between RNA expression and protein expression. Our scRNA-Seq data suggest an increase in *Cd44* and *Icam1* in response to pneumonia in both the adventitial *Cd9^+^Sca1^+^* and alveolar *Pdgfra^+^* fibroblasts, but the flow cytometry analysis showed that PDGFRα*^+^* single-positive fibroblasts, but not Sca1*^+^* single-positive fibroblasts, express CD44 and ICAM1 on the cell surface. These protein findings agree with our conclusion that alveolar fibroblasts responded more robustly to pneumococcal infection; while mRNA of the core response genes was increased in the *Sca1*^+^ cells, this did not translate to an increase in protein levels. Additionally, flow cytometry analysis showed that ICAM1, but not CD44, was strongly expressed by an unexpected Sca1^+^PDGFRα*^+^* double-positive fibroblast population. This population could be a “transitional group” of fibroblasts or a distinct population of cells that have their own response to pneumonia. Previous reports suggest that these double-positive cells may be lipofibroblasts; however, in our dataset, this population lacks other lipofibroblast markers such as *Cd90*/*Thy1*, *Plin2/Adrp*, *Pparg*, and *Cd34* (32, 33, 37). Further studies will be necessary to confirm whether this is a novel fibroblast population; at present, we conclude this population is distinct from alveolar and adventitial fibroblasts.

In conclusion, we demonstrated that matrix fibroblasts, myofibroblasts, pericytes, smooth muscle cells, and mesothelial cells all responded to pneumonia with altered transcriptomes, revealing a core response conserved across cell types as well as distinct mesenchymal cell type–specific responses. The cells from naive and resolved groups overlapped in dimension reduction plots, suggesting mesenchymal cells return to baseline transcriptomes after resolution, unlike other cell types following infections. Of the 5 mesenchymal cell types, fibroblasts were the most abundant mesenchymal cells and could be further segregated into *Pdgfra^+^Npnt^+^Ces1d^+^Col13a1^+^* alveolar fibroblasts and *Cd9^+^Pi16^+^Sca1^+^Col14a1^+^* adventitial fibroblasts. The different subsets of fibroblasts induced similar gene sets, but the alveolar fibroblasts responded more strongly than the adventitial fibroblasts. Versican was induced by both subsets in response to pneumonia but more so in alveolar fibroblasts. We demonstrate that versican expression by these cells is dispensable for neutrophil recruitment, pulmonary edema, and resolution of pneumococcal inflammation and injury. We further demonstrated that while gene expression was increased in both adventitial and alveolar fibroblasts, only alveolar fibroblasts expressed the adhesion molecules CD44 and ICAM1 at the protein level, suggesting distinct responses to infection. These data demonstrate diverse and specialized immune activities of lung mesenchymal cells during pneumonia.

## Methods

### Sex as a biological variable.

All studies involved both sexes and mice at 6 to 14 weeks of age. Sex was considered as a biological variable, and no consistent differences were appreciated. Findings are expected to apply to both sexes.

### Mice.

All animal studies were approved by the Boston University Institutional Care and Use Committee. Six-week-old C57BL6/J (stock 000664), *Pdgfra^tm1.1(cre/ERT2)^* (stock 032770), and *Ifnar1^tm1.2Ees^* (stock 028288) mice were purchased from The Jackson Laboratory. Versican*^tm1.1Cwf^* mice were bred at University of Washington. *Pdgfra^tm1.1(cre/ERT2)^* and *Vcan^tm1.1Cwf^* mice were bred at Boston University School of Medicine. Resulting 6-week-old Cre^–^ and Cre^+^ mice were treated with 2 mg tamoxifen in corn oil daily for 5 days, administered i.p. After 2 weeks of washout, mice were used for experimentation. Mice were housed in a specific pathogen–free environment, on a 12-hour light/12-hour dark cycle, with access to food and water ad libitum. Mice were euthanized using isoflurane overdose, and death was confirmed using pneumothorax before organ collections.

### S. pneumoniae i.t. infection.

Mice were anesthetized via i.p. injection of ketamine and xylazine. To induce pneumonia, the trachea was surgically exposed, and *S*. *pneumoniae* suspended in sterile saline was instilled via a 24-gauge angiocatheter directed toward the left lobe. Heterotypic protection was generated as previously described (12). Mice were infected i.t. with 5 × 10^5^ to 1 × 10^6^ CFU of serotype 19F pneumococcus (Sp19F, strain EF3030) or saline. A week later, mice were exposed again to either saline or Sp19F and allowed to recover for 28 to 35 days. To study acute infection of pneumococcus, mice were challenged i.t. with approximately 1 × 10^6^ CFU of serotype 3 pneumococcus (Sp3, strain ATCC6303) or saline and euthanized 24 to 48 hours after infection.

### Lung cell isolation, flow cytometry, and cell sorting.

The protocol used for preparing single-cell suspensions of mesenchymal cells was modified from Rock et al. (35). The full protocol for cell isolation and staining can be found in Supplemental Methods. Cells were either analyzed using a BD Biosciences LSR II flow cytometer or isolated using a BD Biosciences FACSAria II or Beckman Coulter MoFlo Astrios. Data were analyzed with FlowJo software (BD Biosciences). Gating strategies were based on the use of fluorescence minus one controls.

### Sequencing library construction using the 10x Genomics platform.

scRNA-Seq libraries were prepared per the Single Cell 3′ Reagent Kit User Guide v3 (10x Genomics). Single-cell suspensions (pooled from 3 mice per treatment group; 9 mice total), reagents, and a single Gel Bead containing barcoded oligonucleotides were loaded on a Chromium Controller instrument (10x Genomics) and were encapsulated into a nanoliter-size Gel Bead-in-Emulsion (GEM) using the GemCode platform. Lysis and barcoded reverse transcription of RNAs from single cells was performed. GEMs were harvested and full-length, barcoded cDNA was amplified by PCR to generate sufficient mass for library construction. Enzyme fragmentation, A tailing, adaptor ligation, and PCR were performed to obtain final libraries containing P5 and P7 primers used in the Illumina bridge amplification. Size distribution and molarity of resulting cDNA libraries were assessed via Bioanalyzer High Sensitivity DNA Assay (Agilent Technologies). Sequencing libraries were loaded on a NextSeq 500 (Illumina) instrument according to Illumina and 10x Genomics guidelines, with a 1.4–1.8 μM input and 1% PhiX control library spike-in. Preprocessing and quality control of single-cell data methods can be found in Supplemental Methods. Gene expression data were further analyzed using the online platforms SPRING viewer and Morpheus (36, 51). Transcription factor analysis and pathway enrichment methods can be found in Supplemental Methods.

### qRT-PCR.

RNA was extracted from sorted cells using RNeasy Micro Kit (QIAGEN) as per manufacturer’s protocols. qRT-PCR was performed using the RNA-to-C_T_ kit (Life Technologies). Commercially available predesigned TaqMan gene expression probes for *Vcan* RNA (Mm01283063_m1) and 18S rRNA were used (Applied Biosystems). The quantity of detectable mRNA was calculated by normalizing to 18S rRNA from the respective sample and expressed as fold-change over mRNA levels of controls.

### Wet/dry weights.

Mice were euthanized and left lobes of the lungs were harvested. Wet lung weight was measured in milligrams. Tissues were then incubated at 37°C, and dry lung weight was measured 24 hours later.

### H&E staining and immunohistochemistry.

Mice were euthanized and lungs were perfused with 5 mL 1× PBS. Tracheas were cannulated and lungs were inflated to ~23 cm H_2_O with 10% buffered formalin. Left lobes of fixed tissues were cut into 3 sections and placed in an embedding cassette. Samples were dehydrated and embedded in paraffin. Cooled samples were cut into 5 μm sections. The full protocol for staining, scoring, and immunohistochemistry can be found in Supplemental Methods.

### Human lung biospecimens.

Formalin-fixed, paraffin-embedded postmortem human lung biospecimens were obtained from the Brain and Body Donation Program (https://www.brainandbodydonationregistration.org/) of Banner Sun Health Research Institute in Sun City, Arizona, USA (97).

### Statistics.

All statistical analyses were performed using GraphPad Prism v.9.2.0 software. Error bars represent mean ± SD. A *P* value less than 0.05 was considered significant.

### Study approval.

The study was approved by the Institutional Animal Care and Use Committee (TR202200000005) and the Institutional Review Board (H-32271) of Boston University. Animal procedures were performed with compliance to all relevant ethical regulations for animal testing and research, in the United States, in accordance with the *Guide for the Care and Use of Laboratory Animals* published by the National Institutes of Health (NIH) (National Academies Press, 2011) (after review and approval by the Institutional Animal Care and Use Committee of Boston University or University of Washington). Human participants or their legal representatives gave written informed consent, through an Institutional Review Board, for their participation in the program, including broad consent for sharing of deidentified biospecimens and associated data.

### Data availability.

Values for all data points in graphs are reported in the Supporting Data Values file. scRNA-Seq data for this study are available on the National Center for Biotechnology Information Gene Expression Omnibus under accession number GSE242498. The SPRING plots are available at their respective links listed below.

The full dataset: https://kleintools.hms.harvard.edu/tools/springViewer_1_6_dev.html?client_datasets/Fibroblasts2/Fibroblasts2

Mesenchymal cells in Figure 1: https://kleintools.hms.harvard.edu/tools/springViewer_1_6_dev.html?client_datasets/Fibroblasts2/Mesenchymal_Cells_Only

Matrix fibroblasts in Figure 3: https://kleintools.hms.harvard.edu/tools/springViewer_1_6_dev.html?client_datasets/Fibroblasts2/Naive_and_Inflamed_Fibroblasts

## Author contributions

AMS designed research studies, conducted experiments, acquired and analyzed data, wrote the manuscript, and was responsible for the overall project. JEB and CWF provided materials and contributed to the experimental design and interpretation of results. ATS and FTK aided in conducting leukocyte recruitment experiments. NSE and BEH aided in lung embedding, sectioning, and immunohistochemical experiments. NAC provided histological, immunohistochemical, and pathological expertise. AJ, MB, and JDC provided bioinformatics expertise and aided in data analysis. TGB provided materials and pathological expertise. IMCM, WNG, and CTH assisted with experimentation, including all in vivo animal work. ATS, FTK, KET, MRJ, and LJQ provided intellectual contributions to experiment design and data analysis. JPM designed research studies, procured funding support for experiments, aided in data analysis and interpretation of results, and wrote the manuscript. All authors edited the manuscript.

## Supplementary Material

Supplemental data

Supporting data values

## Version 1 02/25/2025

In-Press Preview

## Version 2 04/08/2025

Electronic publication
