# Supplementary material for: Transcriptomic responses of lung mesenchymal cells during pneumonia
Source: JCI Insight. 2025 Feb 25;10(7):e177084. doi: 10.1172/jci.insight.177084 (PMC11981624; doi:10.1172/jci.insight.177084)
Supplement: Supplemental data [file jciinsight-10-177084-s045.pdf]

Supplemental Methods

Recombinant IFNβ treatment

Mice were anesthetized via i.p. injection of ketamine and xylazine. Recombinant IFNβ was diluted in sterile saline and instilled via a 24-gauge angiocatheter directed toward the left lobe after surgical exposure of the trachea. Mice were given a 50μL bolus of 50,000 units of cytokine and allowed to rest for 0, 4, or 24 hours.

Antibiotic treatment

Mice were treated i.p. with 100mL of 25 mg/mL ceftriaxone (Roche) daily, for 5 consecutive days.

Lung cell isolation, flow cytometry, and cell sorting

The protocol used for preparing single-cell suspensions of lung mesenchymal cells was modified from Rock *et al.* (35). Single-cell suspensions were generated by instilling an enzyme solution of DMEM/F12 (1:1 ratio, Sigma) containing DNase I (33 U/100 mL, Worthington), Type 2 collagenase (45,000 U/100 mL, Worthington), elastase (33 U/100 mL, Worthington), and neutral protease (dispase) (74.4 U/100 mL, Worthington) directly into the lungs prior to harvest. Left lobes of lungs were removed and chopped into small pieces and 4 more mL of enzyme solution were added to digests. Homogenates were incubated for 25 minutes, shaking at 400 rpm at 37°C. Cells were and centrifuged at 2400 rpm for 2 minutes. Media was removed and the pellet was resuspended in DMEM/F12 media containing 0.1% Trypsin-EDTA and DNase I (5 mg/ml). Homogenates were incubated for 25 minutes, shaking at 400 rpm at 37°C. Cells were

passed through a 40 µm cell strainer. Cells were washed and resuspended in 2 mL RBC Lysis buffer (Sigma or BDPharma) for 1 to 2 minutes. Cells were centrifuged at 300xg for 5 minutes at 4°C. Pellets were resuspended in 2 mL FACS buffer (0.4% EDTA and 0.6% FBS in 1x PBS, Millipore-Sigma). Cell concentrations were enumerated on a Luna-FL Dual Fluorescence Cell Counter. $1x10^6$ cells were added to FACS tubes. Cells blocked in 200 µL anti-mouse CD16/32 F_c block (1:25 dilution, clone 93, Biolegend). Cells were incubated for at least 15 minutes on ice while antibody dilutions were prepared. The following antibodies were added to the cells: anti-CD45 PerCP-Cy5.5 (1:200 dilution, Biolegend), anti-CD326 (EpCam) APC-Cy7 (1:200, clone G8.8, BioLegend), anti-CD326 (EpCam) PerCP-Cy5.5 (1:200 dilution, clone G8.8, Biolegend), anti-CD31 (PeCam) Pacific Blue (1:200, clone 390, BioLegend), anti-CD31 (PeCam) PerCP-Cy5.5 (1:200 dilution, clone 390, Biolegend), anti-CD45 PerCP Cy5.5 (1:200 dilution, clone 30-F11, BioLegend), anti-CD140α (PDGFRα) PeCy7 (1:100 dilution, clone APA5, eBioscience), anti-CD54 (ICAM1) APC (1:200 dilution, clone3E2, BD Bioscience), anti-CD44 APC-Cy7 (1:200 dilution, clone 1M7, BD Bioscience), anti-Ly6A/E (Sca1) FITC (1:200 dilution, clone D7, eBioscience). Single cell suspensions were incubated with antibodies for 30 to 60 minutes on ice. Cells were washed with FACS buffer and resuspended in FACS buffer containing the live/dead stain 7AAD (1:60 dilution, BD). Cells were either analyzed using a BD LSR II flow cytometer or isolated using a BD FACSAria II or Beckman Coulter MoFlo Astrios. Data were analyzed with FlowJo software (BD Biosciences). Gating strategies were based on the use of fluorescence minus one (FMO) controls.

Single cell suspensions for isolation of lung leukocytes were generated by first retro-orbitally administering 2 µg anti-CD45 FITC (clone 30-F11, BioLegend) antibody to anesthetized mice. Three minutes later, mice were euthanized. 1ml of anti-CD45 APC (1:100 dilution, clone 104, Biolegend) antibody in sterile saline was instilled directly into the lungs and the antibody was allowed to circulate for 3 minutes. Bronchoalveolar lavage was collected by first removing the first wash of 1-ml antibody. Briefly, lungs were serially lavaged about 9 more times with 1 ml of PBS. The lavage fluid was centrifuged and the cell pellets from each lavage were pooled and used for flow cytometry analysis. Single-cell suspensions were prepared from left lobes as described previously (12). Cells were centrifuged at 300xg for 5 minutes at 4°C. Media was removed and pellets were resuspended in 2 mL FACS buffer. Cell concentrations were enumerated on a Luna-FL Dual Fluorescence Cell Counter. $1x10^6$ cells were added to FACS tubes. Cells were centrifuged at 300xg for 5 minutes at 4°C. Cells were resuspended in zombie Aqua live/dead stain (Biolegend) and incubated away from light for 15 minutes on ice. Samples were centrifuged at 300xg at 4˚C for 5 minutes. Pellets were resuspended in 200 µL anti-mouse CD16/32 F_c-block (1:25 dilution, clone 93, BioLegend). Cells were incubated for at least 15 minutes on ice while antibody dilutions were prepared. Anti-Ly6G APC-Cy7 (1:200 dilution, clone 1A8, BioLegend), anti-CD14 PE (1:200 dilution, clone Sa2-8, eBioscience), and anti-CD45 PerCP-Cy5.5 (1:200 dilution, clone 30-F11, BioLegend) were added to the cells. Single cell suspensions were incubated with antibodies for 30 to 60 minutes on ice. Cells were washed and resuspended with FACS buffer and analyzed. Flow cytometry was performed on LSR II Flow Cytometer (BD Biosciences). Data were analyzed with FlowJo software (BD

Biosciences). Gating strategies were based on the use of Fluorescence minus one (FMO) controls.

Preprocessing and quality control of single-cell data.

The 10X CellRanger v3.1.0 tool was used for demultiplexing, alignment, identification of cells, and counting of unique molecular indices (UMIs). Specifically, the CellRanger mkfastq command was used to demultiplex raw base call (BCL) files generated by Illumina sequencers into FASTQ files. The CellRanger count command was used to perform alignment and create UMI count matrices using parameters –expect-cells=8000. Droplets with at least 500 UMIs underwent further quality control with the SCTK-QC pipeline (1). The median number of UMIs was 3,535, the median number of genes detected was 1,615, the median percentage of mitochondrial reads was 2.72%. Contamination was estimated using decontX (2) in the celda (3) package, and the median contamination score was 0.004 across cells from all samples. Cells with less than 500 counts, less than 300 genes detected, or more than 25% mitochondrial counts were excluded leaving a total of 14,190 cells for the downstream analysis.

Transcription factor (TF) analysis

TF activity was estimated for the mesenchymal data using the decoupleR package (v2.9.1) (4), based on the TFs curated in the CollecTRI (5) regulon resource. TF activity was inferred using the Univariate Linear Model method in decoupleR, following the tutorial. The heatmap of the top 25 TFs with variable activity across cell types was visualized using the pheatmap (v1.0.12) package.

Pathway enrichment

Reactome pathway enrichment analysis for the core and cell type specific genes (both manually curated and unsupervised clustering) was performed based on the ReactomePA (v1.46.0) package (6) using clusterProfiler's (v4.10.0) compareCluster function. The enrichment analysis was performed based on the genes with mappable entrez IDs using clusterProfiler's bitr function, and the organism database was set to "mouse", pvalueCutoff to 0.2, and multiple test correction to Benjamini-Hochberg method. Enriched pathways with adjusted $P < 0.2$ were visualized.

CellChat analysis

Cell-cell communication analysis for the scRNA-seq data was performed using CellChat (v2.1.1) (7) based on the CellChat mouse ligand-receptor database. The default parameters for the analysis were applied following the CellChat tutorial, which included the trimean method to estimate the average gene expression per cell type group and filtering out cell-cell communication which occur in cell types with less than 10 cells. CellChat analysis was performed separately for the pneumonic mesenchymal cells and a combined analysis comparing the interactions between naïve and pneumonic mesenchymal cells. The integrated plotting functions in CellChat were used for visualization.

Versican immunohistochemistry (IHC)

Mice were euthanized and lungs were perfused with 5 mL 1X PBS. Tracheas were cannulated with a blunted 25½ gauge butterfly needle attached to a syringe approximately 23 cm above the base of the base of a ringstand. 10% buffered formalin was added to the syringe and lungs were inflated to ~23 cm H_2O. The trachea was tied off and the heart-lung block removed from the chest cavity. The tissues were placed in a conical containing 10% formalin. Three days later, tissues were transferred to 70% molecular grade ethanol and kept at 4°C overnight. The left lobe of the lung was cut into 3 sections and placed in an embedding cassette. Samples were dehydrated in a graded ethanol series. Tissues were incubated in a 1:1 mix of xylene and paraffin for 1.5 hours at 60°C. Samples were incubated in two changes of paraffin for 45 minutes each at 60°C in a vacuum oven. Lung tissues were removed and embedded in paraffin. Sections were cut at 5 µm for immunohistochemistry (IHC). IHC was performed on a Ventana Discovery Ultra Autostainer (Roche Diagnostics, Indianapolis, IN, USA). Pretreatment was performed with Benchmark Ultra CC1 (Roche), a Tris-based antigen retrieval buffer, at 95°C for 1 hour. Prior to Versican IHC, Glycosamine chains were removed by incubating slides with 0.2 unit/ml chondroitinase ABC (C2905, Sigma-Aldrich, St. Louis, MO, USA) in 18 mM Tris, 1 mM sodium acetate, 1 mg/ml BSA, pH 8.0 in a humidified chamber for 1 hour at 37°C. Pre-dilute HRP polymer antibodies were used for developing all primary antibodies (Vector ImmPress Goat Anti-Rabbit or Mouse IgG (MP-7451 or MP-7452, Vector Laboratories, Newark, CA, USA)) after a protein blocking step with Akoya Opal Diluent/Block (ARD1001EA, Akoya Biosciences, Marlborough, MA, USA). Primary and secondary antibody complexes were developed using Opal fluorophores (Akoya Biosciences). Slides were counterstained with Akoya

Biosciences Spectral DAPI and coverslipped with Prolong Gold Antifade Mountant (P36930, Invitrogen, Waltham, MA, USA). A summary of the monoplex Versican and multiplex assay is provided in Supplemental Table 7. Whole slide images were acquired and spectrally unmixed with a PhenoImager HT TM Automated Quantitative Pathology Imaging System (Akoya Biosciences). For quantification of versican localization, perivascular and peribronchovascular boundaries were outlined using QuPath software.

H&E staining and Immunohistochemistry

Mice were euthanized and lungs were perfused with 5 mL 1X PBS. Tracheas were cannulated with a blunted 25½ gauge butterfly needle attached to a syringe approximately 23 cm above the base of the base of a ringstand. 10% buffered formalin was added to the syringe and lungs were inflated to ~23 cm H_2O. The trachea was tied off and the heart-lung block removed from the chest cavity. The tissues were placed in a conical containing 10% formalin. Three days later, tissues were transferred to 70% molecular grade ethanol and kept at 4°C overnight. The left lobe of the lung was cut into 3 sections and placed in an embedding cassette. Samples were dehydrated in a graded ethanol series. Tissues were incubated in a 1:1 mix of xylene and paraffin for 1.5 hours at 60°C. Samples were incubated in two changes of paraffin for 45 minutes each at 60°C in a vacuum oven. Lung tissues were removed and embedded in paraffin. Samples were cooled overnight at 4°C and sectioned into 5μM sections the next day. Sections were transferred to a warm water bath and placed on charged slides. Slides were incubated at 37°C overnight to dry and fix tissues to the slides. For H&E staining, slides were deparaffinized in xylene and rehydrated in a reverse graded ethanol series.

Slides were stained with hematoxylin and eosin prior to dehydration in a graded ethanol series and subsequent coverslip mounting. A detailed scoring system for lung inflammation and injury is outlined in Supplemental Table 8. For the human autopsy sample, a Ventana Discovery Ultra (Roche, Basel, Switzerland) tissue autostainer was used for multiplex fluorescent immunohistochemistry (mfIHC). Species-specific secondary antibodies conjugated to horseradish peroxidase (HRP) were utilized to covalently bind fluorophores to tissues. Multiplexing was achieved using tyramide signaling amplification (TSA) whereby antigens were developed in an iterative approach to covalently bind Opal fluorophores (Akoya Biosciences, Marlborough, MA) to tyrosine residues in tissue sections, with subsequent heat stripping of primary-secondary antibody complexes until all antibodies were developed. Before mfIHC was performed, each antibody was individually optimized using a single-plex IHC assay using an appropriate positive control tissue known to possess the antigen under investigation. Negative controls included lung autopsy samples that had no significant pulmonary disease. Optimization experiments were performed to determine ideal primary antibody dilution, sequential order of antibody development, assignment of each primary antibody to an Opal fluorophore, and fluorophore titration. All Opal TSA-conjugated fluorophore reactions took place for 20 minutes. Fluorescent slides were counterstained with spectral DAPI (Akoya Biosciences) for 16 minutes before being mounted with ProLong gold antifade (ThermoFischer, Waltham, MA). Antibodies utilized in 5-plex analysis included: Versican, CD68, myeloperoxidase (MPO), CD31, smooth muscle actin. Primary antibodies were developed with a secondary goat anti-rabbit or anti-mouse HRP polymer antibody (Vector Laboratories, Burlingame, CA) for 20 minutes at 37°C.

Detailed optimization parameters outlined in Supplemental Table 7. Images were acquired using a Vectra Polaris whole slide imager (Akoya Biosciences).

Supplemental Figures and Tables

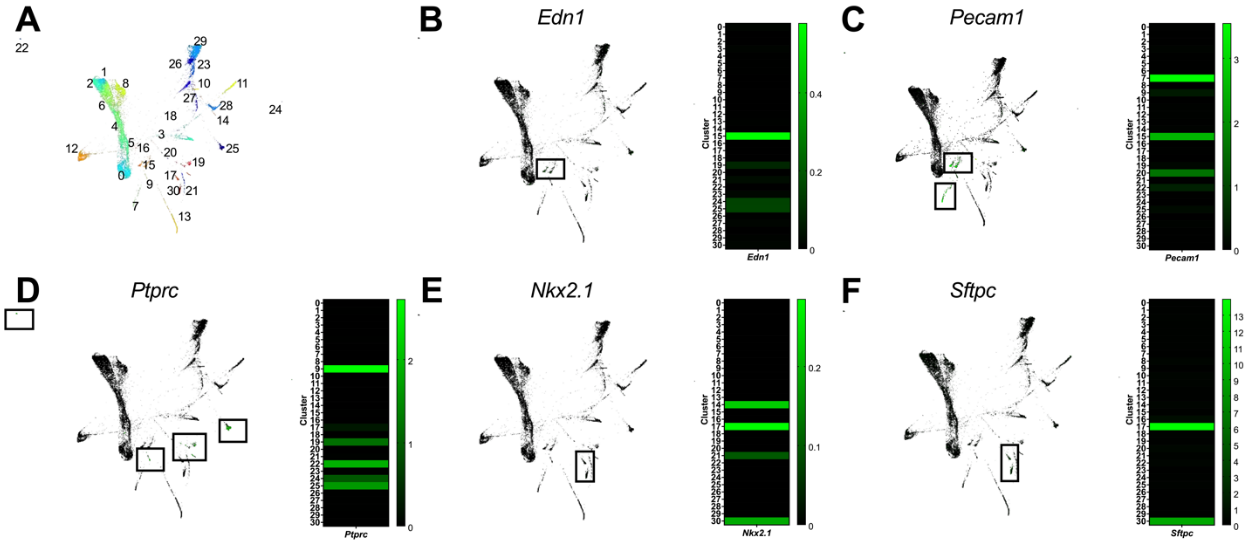

Supplemental Figure 1. Non-mesenchymal cells were removed from single cell RNA sequencing dataset. (A) SPRING nearest-neighbor plots from single-cell RNA sequencing of CD45- CD326- CD31- cells collected from naïve, pneumonic, and resolved lungs collected 24 hours after i.t. instillations of sterile saline or *S. pneumoniae* show 31 distinct Louvain clusters. (B) SPRING plot and heatmap showing the normalized RNA level of *Edn1* is strongest in cluster 15. (C) SPRING plot and heatmap showing the normalized RNA level of *CD31* aka *Pecam1* is strongest in clusters 7, 15, and 20. (D) SPRING plot and heatmap showing the normalized RNA level of *CD45* aka *Ptprc* is strongest in clusters 9, 19, 22, 24, and 25. (E) SPRING plot and heatmap showing the normalized RNA level of *Nkx2.1* is strongest in clusters 14, 17, and 30. (F) SPRING plot and heatmap showing the normalized RNA level of *Sftpc* is strongest in clusters 17 and 30.

Manual Curation

Unsupervised Clustering

Angptl4	*Bst2*	*Rsad2*
Ccl2	*H2.T22*	*Ifi44*
Cp	*Cxcl1*	*Pim1*
Eif6	*Birc2*	*Gbp9*
F830016B08Rik	*Sod2*	*Tnfaip3*
Fas	*Cxcl10*	*Gars*
Gbp5	*Iigp1*	*Chmp4b*
Gbp7	*Marchf5*	*Ogfr*
Gm4951	*Irgm1*	*Lgals9*
H2.T23	*Nfkbia*	*Eif2ak2*
Hif1a	*Irf7*	*Usp18*
Igtp	*Ifit3*	*Cd274*
Irf1	*Ifi35*	*Mitd1*
Lcn2	*Phf11d*	*Irgm2*
Lgals3bp	*Ifit1*	*Irf9*
Mif	*Isg15*	*Parp9*
Mrpl52	*Psmb8*	*Myd88*
Mt1	*Psmb10*	*Oasl2*
Mt2	*Xaf1*	*Parp12*
Nme1	*Herc6*	*Shisa5*
Parp14	*Ifi47*	*Ube2l6*
Pnp	*Psmb9*	*Snx10*
Ppa1	*Rtp4*	*Rab5c*
Rnf213	*Samhd1*	
Saa3	*Igtp*	
Stat1	*Gbp2*	
Susd6	*Gpb3*	
Tap1	*Psme1*	
Tap2	*Psme2*	
Timp1		
Vmp1		

Supplemental Table 1. Core genes induced at least 2-fold in all mesenchymal cell subsets after bacterial infection with *S. pneumoniae,* revealed by either manual curation or unsupervised hierarchical clustering. Grey denotes genes discerned by both clustering methods.

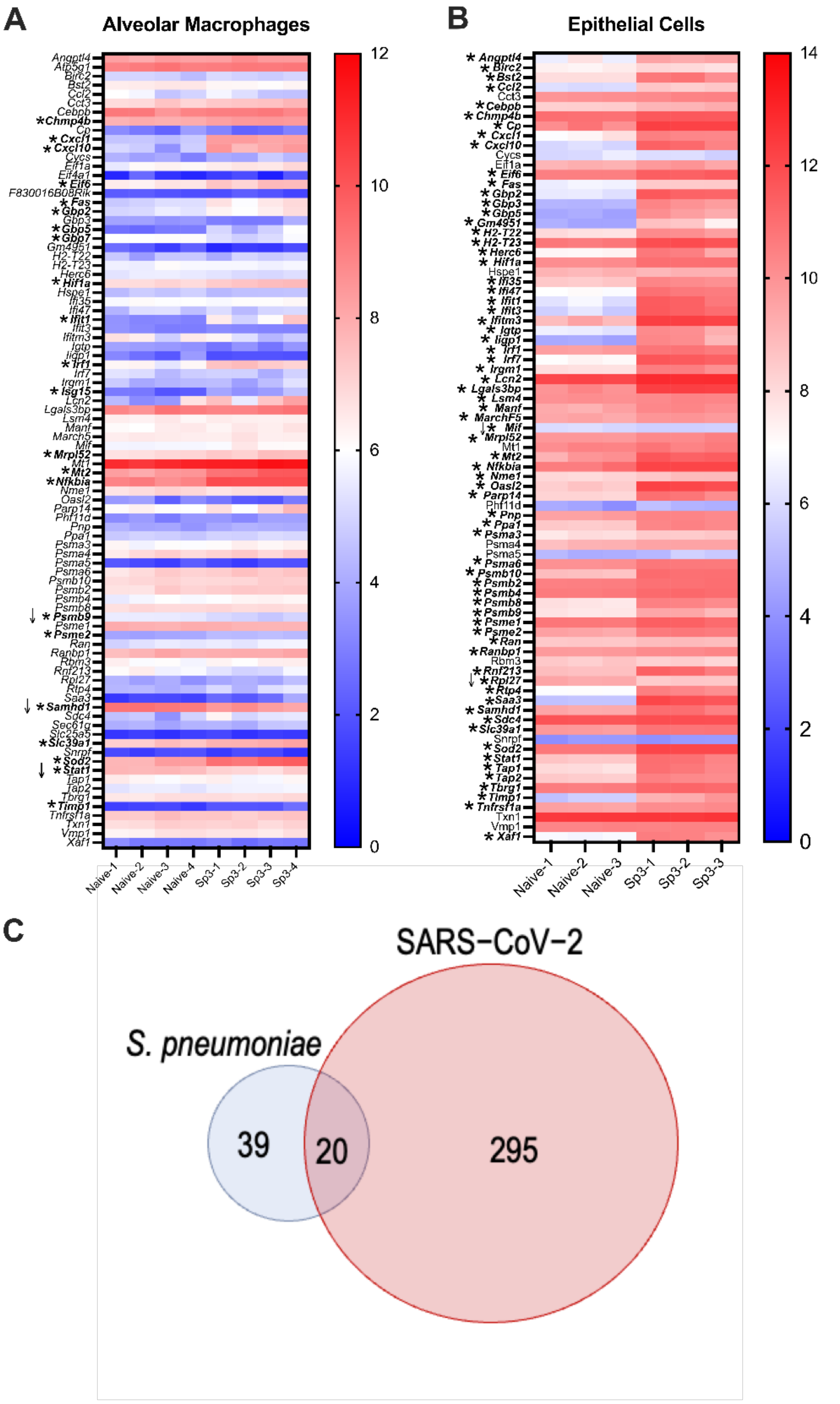

Supplemental Figure 2. Heatmaps showing log2 expression levels of the 59 mesenchymal cell core response genes in (A) alveolar macrophages and (B) epithelial cells during pneumococcal pneumonia. Asterisks (*) indicate significantly changed gene responses in infected samples compared to naïve. Down arrows (↓) indicated down regulation of genes in response to pneumonia. (C) Venn diagram comparing genes upregulated in *S. pneumoniae* (blue) and/or SARS-CoV-2 (red) pulmonary infections.

Manual Curation		Unsupervised Clustering
Gm20186	*Anxa8*	*Apol9a*
	Dmkn	*Apol9b*
	Pvrl2	*Gstp1*
	Chil1	*Polr3k*
	Prg4	*Shb*
	Cldn10	*Atp1a1*
	Krt18	*Arhgdib*
		Cxadr
		Bicd1
		Plet1
		Gm20186
		Efna5
		Krt8
		Slpi
		Wwc1
		Slc9a3r1
		Plscr1
		Mpp6
		Has1
		Htra2
		Lbp
		Pdpn
		Nnmt
		Scamp2
		Tubb2a
		Echs1
		Glrx
		Mettl9
		Ppp1r14b
		Taf10
		Serp1
		Sra1
		Mgst1
		Ptgis
		Anxa1
		Smpd1
		Tmem147
		S100a16
		Zcchc11
		Gm12840

Supplemental Table 2. Specific genes induced at least 2-fold in only mesothelial cells after bacterial infection with *S. pneumoniae,* revealed by either manual curation or unsupervised hierarchical clustering. Grey denotes genes discerned by both clustering methods.

Manual Curation

Gja4
Uba7
Cnn3
Ttc39b

Arid5a
Tmem140
Cd40
Irf8
Nckap5
Batf2
Slc15a3
Flt1
Slfn5
Sema4c
Rhoj

Unsupervised Clustering

Agrn
Adam9
Grina
Sp100
Gm26917
Zc3h7a
Gdf15
Camkk2
Ddit3
D17Wsu92e
Gna13
Azi2
Ptpn1
Znrf1
Jak2
Cxcl9
Ripk1
Gtpbp2
Trim56
Pgf

Supplemental Table 3. Specific genes induced at least 2-fold in only pericytes after bacterial infection with *S. pneumoniae,* revealed by either manual curation or unsupervised hierarchical clustering. Grey denotes genes discerned by both clustering methods.

Manual Curation		Unsupervised Clustering
	Cxcl13	
	Enc1	
	Hp	
	Cxcl5	
Serpina3m	*Ptx3*	
Egfr	*Ifi205*	
Csf3	*Mpeg1*	
Slfn2	*Serpina3f*	
Nop56	*Ptges*	
Lcn2	*Lox*	
Timp1	*Serpina3n*	n/a
Plac8	*Fxbp11*	
Saa3	*Vcan*	
Pum3	*Sphk1*	
Cstb	*Tnc*	
Tnc	*Slc16a1*	
	Col4a1	
	Col4a2	
	Dram1	
	Prdx6	

Supplemental Table 4. Specific genes induced at least 2-fold in only matrix fibroblasts after bacterial infection with *S. pneumoniae,* revealed by either manual curation or unsupervised hierarchical clustering. Grey denotes genes discerned by both clustering methods.

**Manual Curation**		**Unsupervised Clustering**
Tnfaip6<br>*Vimp*<br>*Dnajb11*<br>*Psmd14*<br>*Mapkapk2*<br>*Lmo4*<br>*Gclm*	n/a	n/a

Supplemental Table 5. Specific genes induced at least 2-fold in only myofibroblasts after bacterial infection with *S. pneumoniae,* revealed by either manual curation or unsupervised hierarchical clustering. Grey denotes genes discerned by both clustering methods.

Manual Curation	Both	Unsupervised Clustering
Cd200	*Fgl2*	*Hmgn3*
Vcam1	*Fabp4*	*X2200002D01Rik*
Pde1a	*Hspb1*	*Hacd1*
Isyna1	*Itih4*	*Pitpna*
Cdo1		*Mrps17*
Pde4b		

Supplemental Table 6. Specific genes induced at least 2-fold in only smooth muscle cells after bacterial infection with *S. pneumoniae,* revealed by either manual curation or unsupervised hierarchical clustering. Grey denotes genes discerned by both clustering methods.

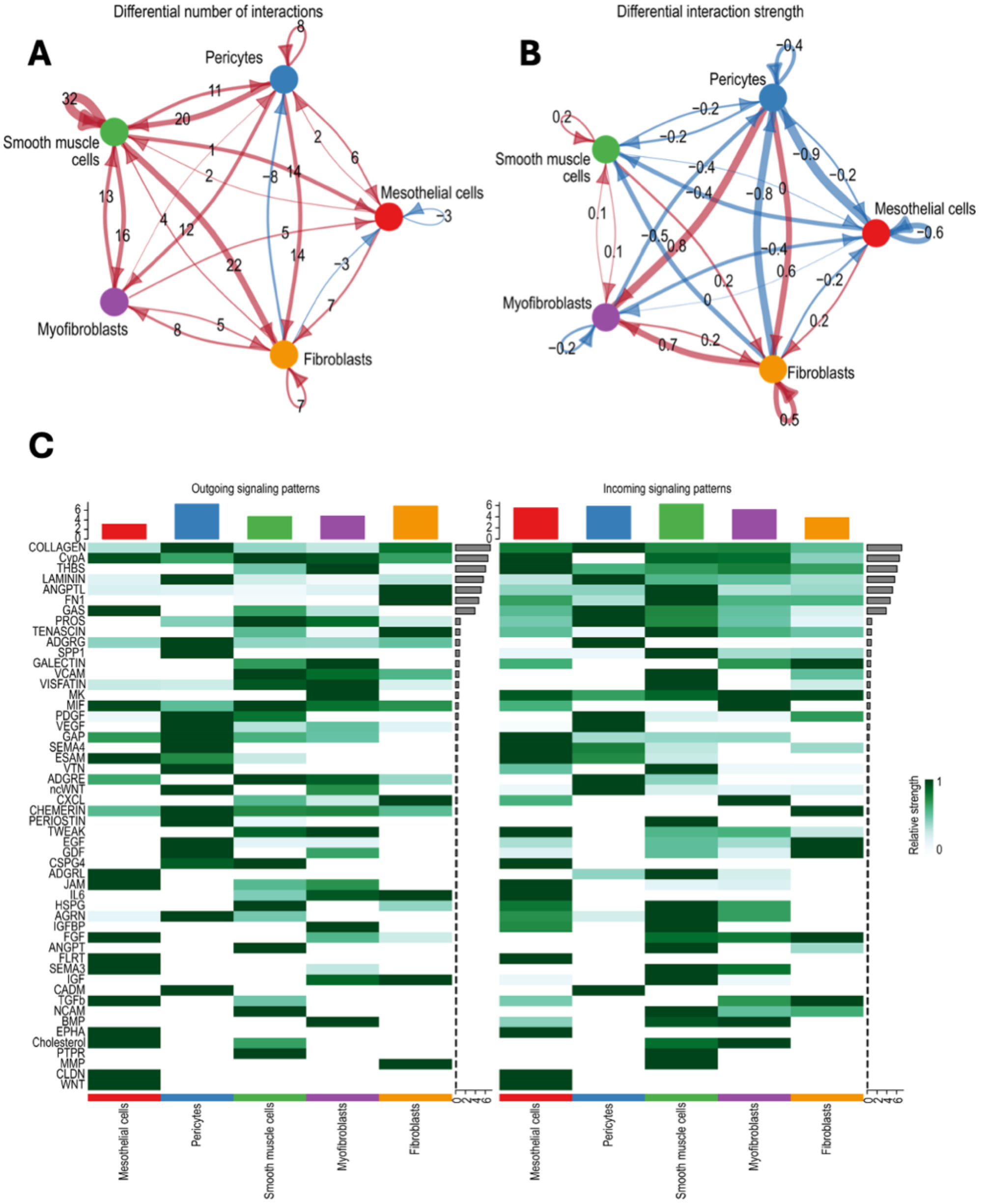

Supplemental Figure 3. (A) Plot showing differential number of interactions and (B) interaction strength among the mesenchymal cell types between naïve and pneumonic mice. Circles represent each cell type and lines connecting the circles (edges) represent the interaction between cell types. The color of the edges represents increased (red) or decreased (blue) interaction between the cell types in pneumonic mice versus naïve mice. The numbers indicate the number of interactions or interaction strength. (C) Heatmap of signaling pathways mediating the outgoing and incoming cell-to-cell communication between mesenchymal cells in pneumonic mice. The top bar plot indicates the total signaling strength within a cell type. The bar plot on the side represents the total signaling strength of each pathway across cell types.

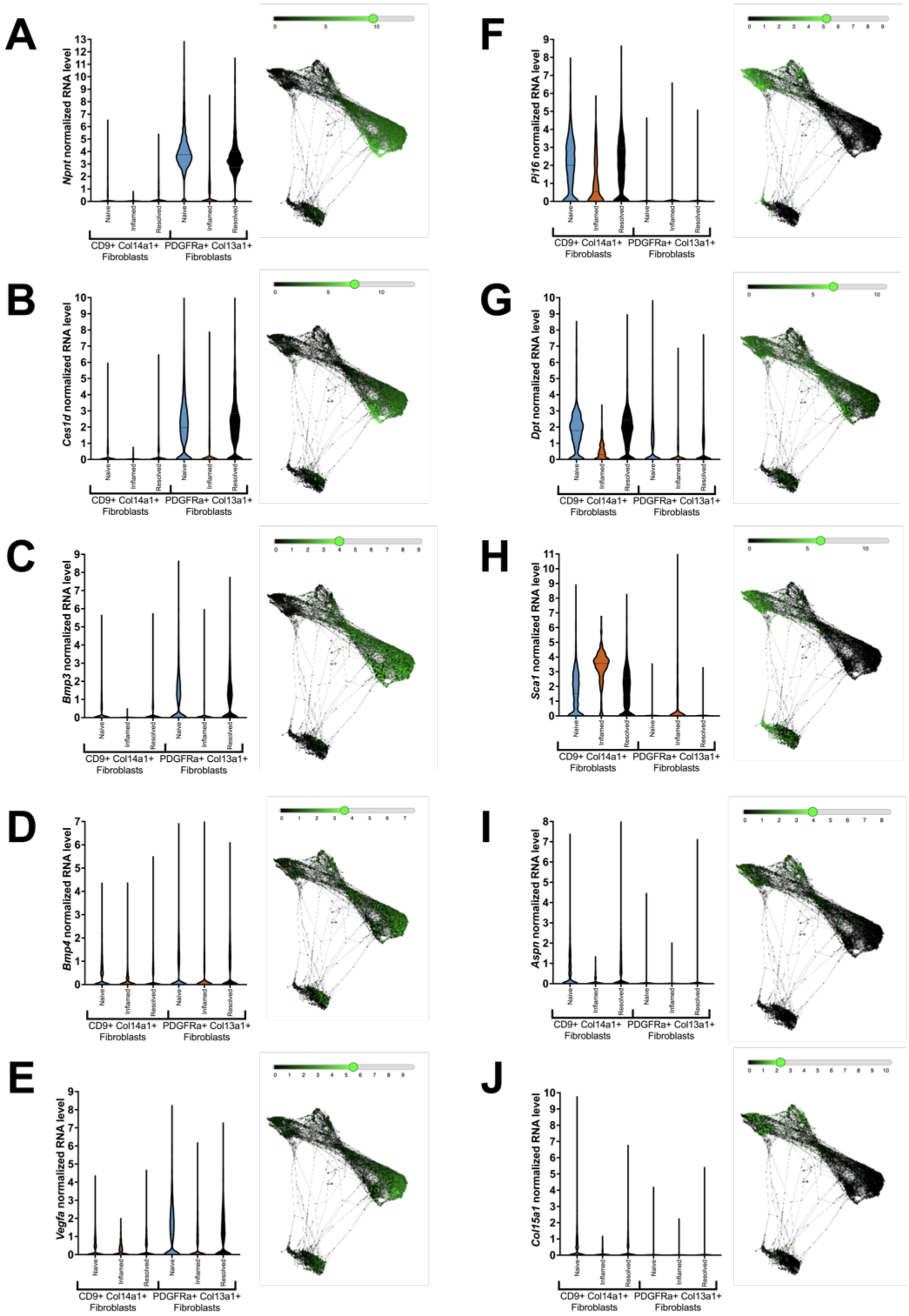

Supplement Figure 4. (A-E) Violin and SPRING plots showing normalized RNA levels of *Npnt, Ces1d*, *Bmp3*, *Bmp4, Vegfa* which have all been associated with alveolar fibroblasts. (F-J) Violin and SPRING plots showing normalized RNA levels of *Pi16*, *Dpt*, *Sca1*, *Aspn*, and *Col15a1* which are genes associated with adventitial fibroblasts that are found in all tissue types.

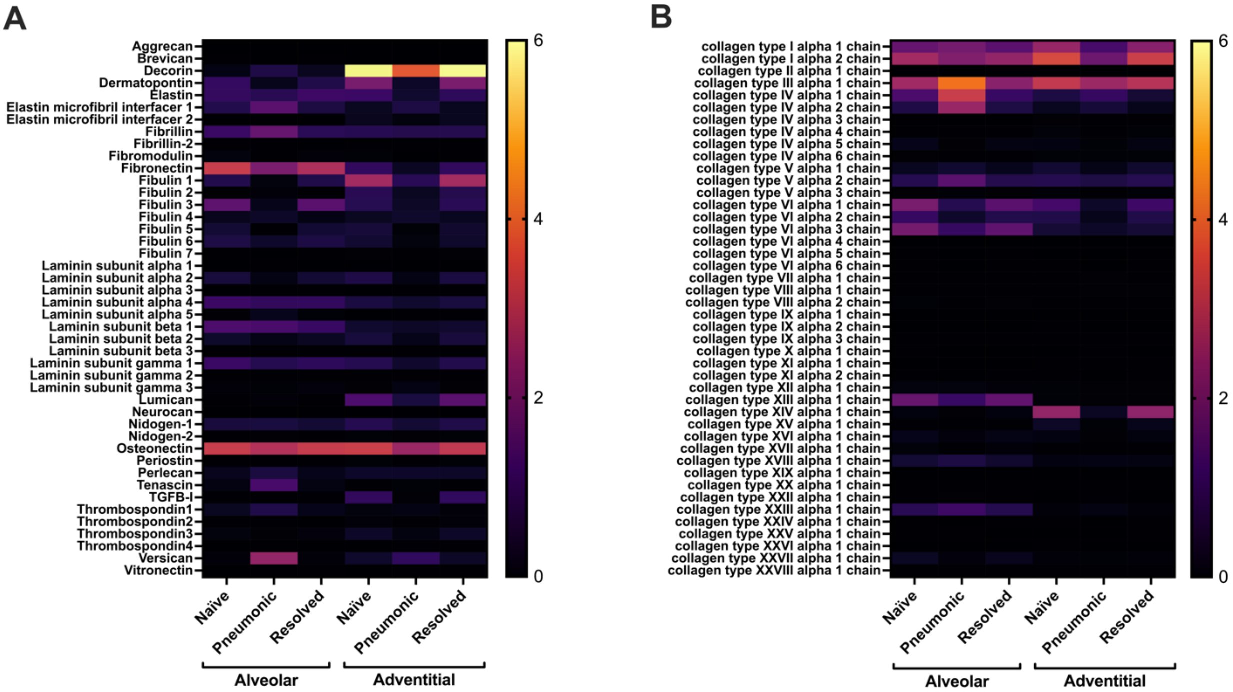

Supplement Figure 5. Extracellular matrix protein (A) and collagen (B) normalized RNA levels in alveolar and adventitial fibroblast cells isolated from naïve, pneumonic, and resolved lungs.

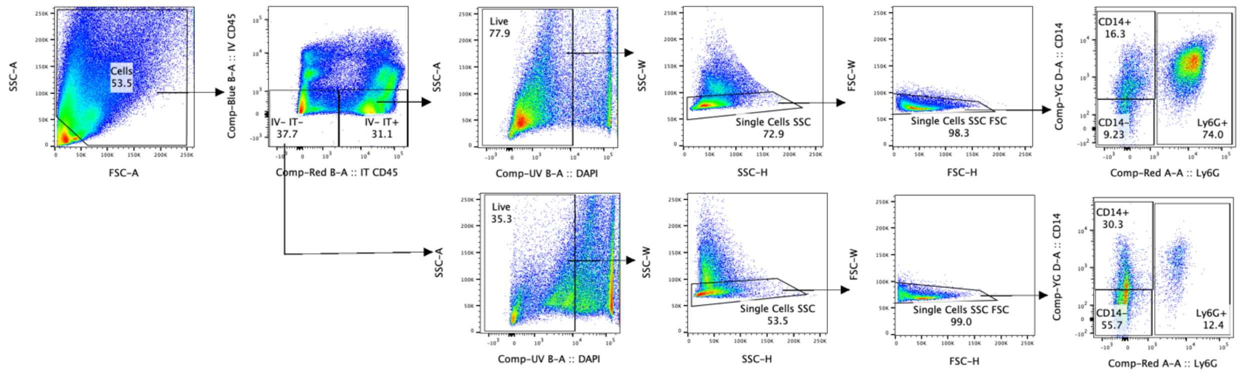

Supplement Figure 6. Gating strategy for leukocyte localization flow cytometry experiment in PDGFRA^{CreERT2} / Versican$^{tm1.11Cwf}$ or PDGFRA$^{Cre\text{-}null}$ / Versican$^{tm1.1Cwf}$ mice 24 hours after i.t. instillations of *S. pneumoniae.* Debris was gated out using forward versus side scatter, then airspace cells (IT+) and non-airspace (IT-) CD45 cells were gated using the extravascular and intratracheal staining of CD45. IT- cells were further analyzed using the final CD45 staining, to identify CD45+ cells in the lung tissue. Airspace (IT+) and interstitial (IT- CD45+) were then further analyzed using the live/dead stain and doublet exclusion criteria. Lastly, cells were analyzed for Ly6G (neutrophils) and CD14 (monocyte/macrophage) expression.

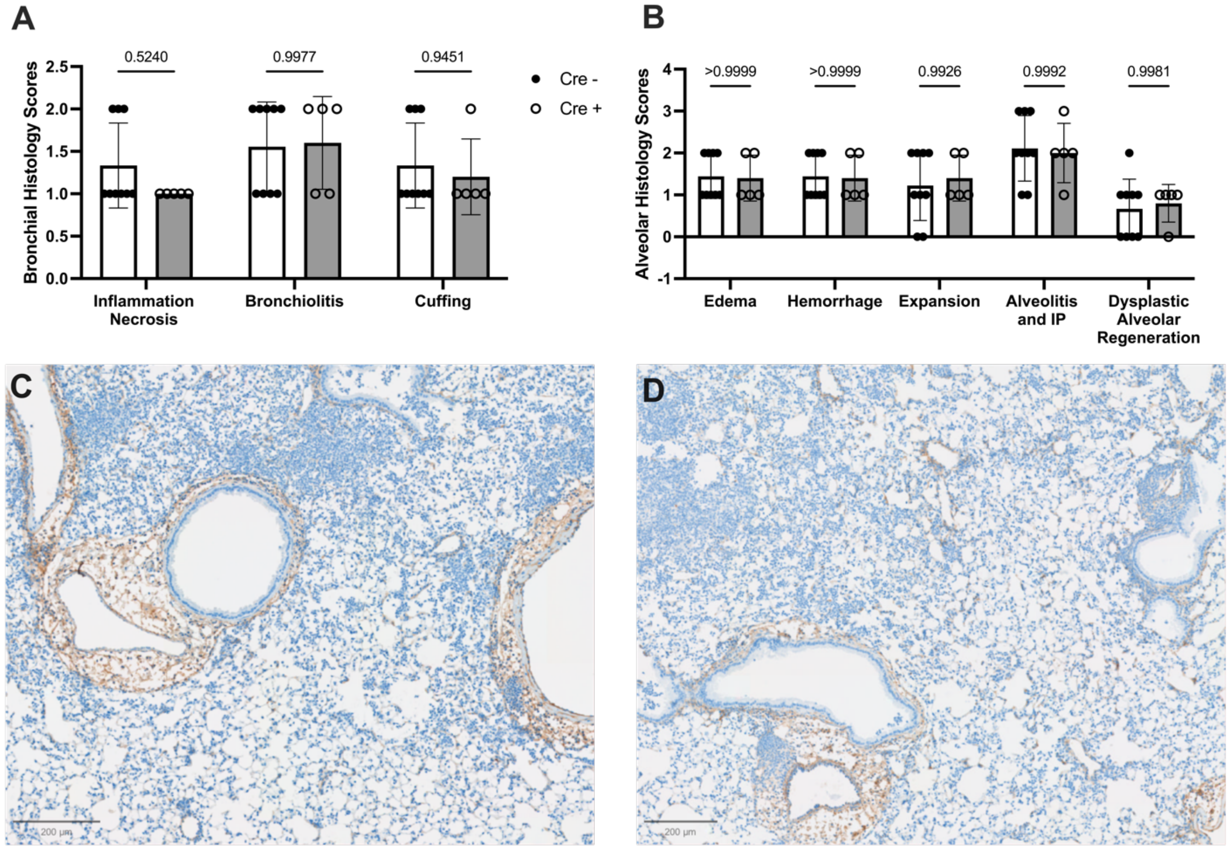

Supplemental Figure 7. Histological analysis of H&E-stained lung sections from PDGFRA$^{Cre\text{-}null}$ / Versican$^{tm1.1Cwf}$ (Cre-) and PDGFRA^{CreERT2} / Versican$^{tm1.11Cwf}$ (Cre+) mice after a lethal dose of *S. pneumoniae* and subsequent antibiotic treatment shows no remarkable difference in (A) bronchial and (B) alveolar inflammation and injury. Significance was determined by two-way ANOVA followed by Šidák's multiple comparisons test. Versican IHC staining shows no remarkable difference in location of versican in (C, n=11) Cre- or (D, n=3) Cre+ mice 24 hours after i.t. instillations of *S. pneumoniae.* Scale bars represent 200 µm.

Versican IHC					
Protein specificity	Host (clone)	Dilution	Vendor	Incubation	Chromogen
Versican	Rabbit (EPR23135-58)	1:75	Abcam	1 hour, 37°C	DAB

Multiplexed IHC							
Sequence	Protein specificity	Host (clone)	Dilution	Vendor	Incubation	OpalTM Fluor. (Akoya Biosci-ences)	Fluor. Dilution
1	Versican	Rabbit (EPR23 135-58)	1:75	Abcam	1 hour, 37°C	520 (green)	1/100
2	Myeloper oxidase (MPO)	Rabbit (E1E7I)	1:200	Cell Signaling Technology	40 minutes, 37°C	480 (cyan)	1/125
3	Smooth muscle actin (SMA)	Rabbit (D4K9N)	1:200	Cell Signaling Technology	40 minutes, 37°C	570 (yellow)	1/200
4	CD31	Mouse (BC2)	1:50	Biocare Medical	1 hour, 37°C	620 (red)	1/100
5	CD68	Mouse (KP1)	1:60	LSBio	40 minutes, 37°C	690 (purple)	1/150

Supplemental Table 7. Immunohistochemisty (IHC) optimization. Antigen retrieval for both multiplex panels included 32 minutes of CC1 (Tris based) at 95°C with subsequent antibody stripping iterations of 24 minutes of CC2 (Citrate based) at 100°C. Fluorophore (Fluor.)

	Numerical Score			
Parameter measured	0	1	2	3
Inflammatory Cells and Necrotic Debris in the Bronchial Lumens	None	Mild	Moderate	Severe
Bronchiolitis	None	Early epithelial changes with occasional loss of cilia	Mild flattening with loss of cilia and club cells	Flattened epithelium with epithelial loss (necrosis)
Peribronchiolar leukocyte cuffing	None	Mild, loosely formed cuffs of leukocytes	Moderate, well-formed cuffs of leukocytes	Prominent thick well-formed cuffs of leukocytes
Perivascular leukocyte cuffing	None	Mild, loosely formed cuffs of leukocytes	Moderate, well-formed cuffs of leukocytes	Prominent thick well-formed cuffs of leukocytes
Perivascular Edema	None	Minimal amounts of edema	Moderate amounts of edema	Prominent edema with dilated lymphatics
Alveolar Edema	None	Mild	Moderate	Severe
Alveolar Hemorrhage	None	Mild	Moderate	Severe
Alveolar Expansion	None	Mild	Moderate	Severe
Alveolitis and Interstitial pneumonia	None	Mild, focal to multifocal IP	Moderate, locally extensive to multifocal IP	Severe, multifocal to coalescing IP
Dysplastic Alveolar Regeneration	None	Mild, focal to multifocal	Moderate, locally extensive to multifocal	Severe, multifocal to coalescing

Supplemental Table 8. Histological scoring system for inflammation and injury in H&E-stained lung sections.

Supplemental References

1. Hong R, et al. Comprehensive generation, visualization, and reporting of quality control metrics for single-cell RNA sequencing data. *Nat Commun*. 2022;13(1).

2. Yang S, et al. Decontamination of ambient RNA in single-cell RNA-seq with DecontX. *Genome Biol*. 2019.

3. Wang Z, et al. Celda: A Bayesian model to perform co-clustering of genes into modules and cells into subpopulations using single-cell RNA-seq data. *NAR Genom Bioinform*. 2022;4(3): lqac066.

4. Badia IMP, et al. decoupleR: ensemble of computational methods to infer biological activities from omics data. *Bioinform Adv*. 2022;2(1):vbac016.

5. Müller-Dott S, et al. Expanding the coverage of regulons from high-confidence prior knowledge for accurate estimation of transcription factor activities. *Nucleic Acids Res*. 2023;51(20):10934-49.

6. Yu G, He QY. ReactomePA: an R/Bioconductor package for reactome pathway analysis and visualization. *Mol Biosyst*. 2016;12(2):477-9.

7. Jin S, et al. Inference and analysis of cell-cell communication using CellChat. *Nat Commun*. 2021;12(1):1088.
